# Design, Synthesis, and Biological Activities of Novel Pyrazole Oxime Compounds Containing a Substituted Pyridyl Moiety

**DOI:** 10.3390/molecules22060878

**Published:** 2017-05-25

**Authors:** Cuili Chen, Jia Chen, Haiying Gu, Ning Bao, Hong Dai

**Affiliations:** 1 College of Chemistry, Chemical Engineering and Materials Science, Soochow University, Suzhou 215123, China; cleancucumber@163.com; 2 School of Public Health, Nantong University, Nantong 226019, China; ningb2000@yahoo.com; 3 College of Chemistry and Chemical Engineering, Nantong University, Nantong 226019, China; 15642891665@163.com

**Keywords:** pyrazole oxime, pyridyl, synthesis, biological activity

## Abstract

In this paper, in order to find novel biologically active pyrazole oximes, a series of pyrazole oxime compounds bearing a substituted pyridyl unit were prepared. Bioassays showed that some target compounds were found to have good acaricidal activity against *Tetranychus cinnabarinus* at a concentration of 500 μg/mL, compound **9q** especially displayed potent acaricidal activity against *T. cinnabarinus* when the concentration was reduced to 100 μg/mL. Interestingly, most target compounds possessed excellent insecticidal activities against *Oriental armyworm* at 500 μg/mL. Moreover, some compounds were active against *Aphis medicaginis* and *Nilaparvata lugens* at 500 μg/mL. Additionally, compounds **9b**, **9g**, **9l**, **9p**, **9q**, **9r**, **9s**, **9t**, **9u**, and **9v** displayed significant antiproliferative activities against HepG2 cells with IC_50_ values of 1.53–17.27 μM, better than that of the control 5-fluorouracil (IC_50_ = 35.67 μM).

## 1. Introduction

**Figure 1:**
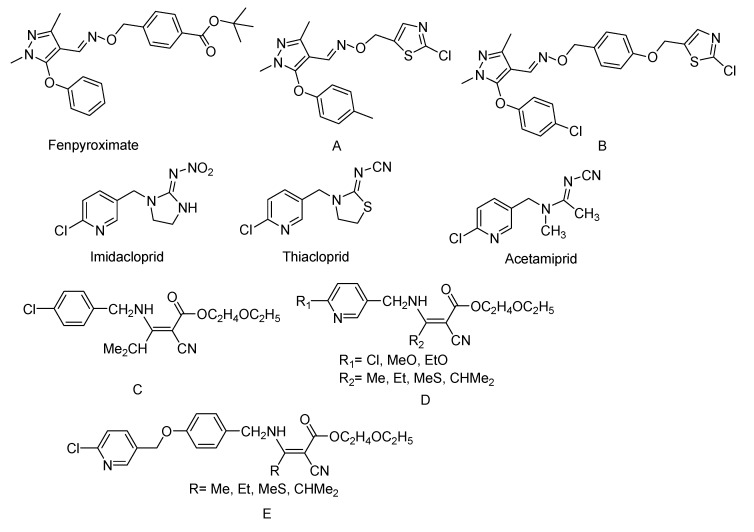
Structures of fenpyroximate, imidacloprid, thiacloprid, acetamiprid, and compounds **A**–**E**.

In the past few decades, heterocycles play a significant role in the research of agricultural and medicinal chemistry. Pyrazole oxime is a vital five-membered aromatic heterocycle. A lot of pyrazole oxime derivatives have drawn considerable attention for their extensive biological activities including insecticidal [1], acaricidal [2], fungicidal [3], antitumor [4], and antiviral activity [5]. For example, fenpyroximate (Figure 1), a potent acaricide carrying a pyrazole oxime unit, is used to control some important phytophagous mites such as *Tetranychus urticae* Koch and *Polyphagotarsonemus latus* Banks [6]. After it appeared on the market in 1991, many chemists began to study the structural modification of fenpyroximate. Recently, Zou et al. reported that thiazole-containing pyrazole oxime compound **A** (Figure 1) displayed good insecticidal activity [7], Dai et al. obtained pyrazole oxime compound **B** (Figure 1) bearing a benzyloxy-linked thiazole unit exhibiting interesting insecticidal activity besides good acaricidal activity [8]. Park et al. reported some Fenpyroximate analogues possessing potential anticancer properties against HepG2 (human hepatoma) cells [9], and Dai et al. also found some 1,2,3-thiadiazole-containing pyrazole oxime derivatives displaying satisfactory antitumor activities against Panc-1 (human pancreatic carcinoma) and SGC-7901 (human gastric cancer) cells [10]. Therefore, pyrazole oxime compounds became a focus of chemical and pharmaceutical research.

On the other hand, the pyridyl ring is another important six-membered aromatic heterocycle containing one nitrogen atom, which plays a vital role in lots of biologically active compounds [11,12,13,14]. Many pyridine-based derivatives are found to possess broad spectrum biological activities such as fungicidal [15], insecticidal [16,17,18], herbicidal [19], antiviral [20], and anticancer activities [21]. For instance, imidacloprid, thiacloprid, and acetamiprid (Figure 1), well-known pyridyl-containing neonicotinoid insecticides, are currently used in the fields of crop protection and animal health due to their good insecticidal activities and low mammalian toxicity [22]. Recently, Wang et al. reported the preparation of novel 2-cyanoacrylates **D** (Figure 1) and replaced the substituted phenyl ring of compound **C** (Figure 1) with a substituted pyridyl group, resulting in better biological activity and broader bioactivity spectra [23]. More recently, Wu et al. found that some 2-cyanoacrylates **E** (Figure 1) carrying a benzyloxy-linked pyridyl unit also indicated wonderful bioactivities [24]. So we have reason to believe that the substituted pyridyl moiety can be used as a significant skeleton in exploring novel bioactive molecules.

**Figure 2:**
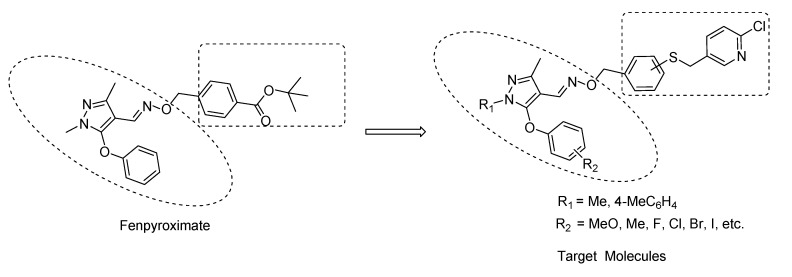
The design of the title molecules.

Motivated by the above viewpoints, we conceived that replacement of the esterified phenyl ring of fenpyroximate with a substituted pyridine group might produce some new compounds with good biological activities (Figure 2). In this research, we report the synthesis and bioactivities of novel pyrazole oximes containing a substituted pyridyl moiety.

## 2. Results and Discussion

### 2.1. Chemistry

**Scheme 1:**
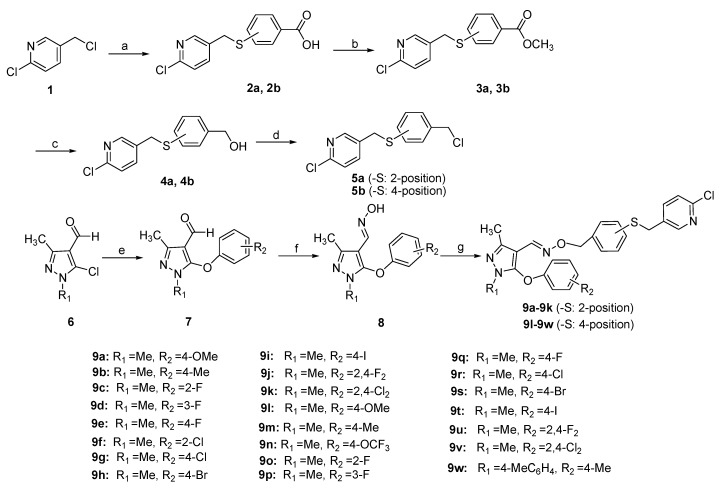
Synthesis of the target compounds **9a**–**9w**. *Reagents and conditions:* (a) 2-mercaptobenzoic acid or 4-mercaptobenzoic acid, potassium hydroxide, ethanol, reflux for 10 h, 65% for **2a**, 70% for **2b**; (b) methanol, concentrated sulfuric acid, reflux for 8 h, 75% for **3a**, 83% for **3b**; (c) LiAlH_4_, tetrahydrofuran, 0 °C for 10 min, r.t. for 2 h, 63% for **4a**, 71% for **4b**; (d) thionyl chloride, dichloromethane, 0 °C for 10 min, r.t. for 3 h, 73% for **5a**, 80% for **5b**; (e) substituted phenols, potassium hydroxide, DMF, 40 °C for 6 h, then 100 °C for 12–20 h, 58–80% for **7**; (f) hydroxylamine hydrochloride, potassium hydroxide, methanol, reflux for 8–15 h, 56–77% for **8**; (g) compounds **5**, potassium carbonate, cesium carbonate, acetonitrile, reflux for 8–17 h, 53–75% for **9**.

As indicated in Scheme 1, 23 pyrazole oxime compounds carrying substituted pyridine moiety were successfully prepared. Using potassium hydroxide as the base, compound **1** was easily reacted with 2-mercaptobenzoic acid or 4-mercaptobenzoic acid to give compound **2**. Further reaction with methanol, under acidic condition, afforded compound **3** in satisfactory yields. The next reaction with LiAlH_4_ produced compound **4** in good yields. Then chlorination of compound **4** provided the key intermediate **5** successfully. The condensation of compound **6** with different substituted phenols under basic conditions afforded 5-aryloxy substituted pyrazole carbaldehyde (**7**), which was then transformed to 5-aryloxy substituted pyrazole oximes (**8**) by the treatment with hydroxylamine hydrochloride. Finally, the reaction of the key intermediate **5** with oximes **8**, under cesium carbonate promoting conditions, provided the designed compounds **9a**–**9w** in good yields. The title compounds have all been structurally confirmed through ^1^H NMR, ^13^C NMR and elemental analyses.

### 2.2. Biological Activities

#### 2.2.1. Acaricidal Activities and Insecticidal Activity

The synthesized compounds **9a**–**9w** were tested for acaricidal activity against *Tetranychus cinnabarinus* and insecticidal activities against *Oriental armyworm*, *Aphis medicaginis* and *Nilaparvata lugens*. Abamectin and Fenpyroximate were used as the positive controls, respectively. As displayed in Table 1, some of the target compounds showed good acaricidal activity against *T. cinnabarinus* at a concentration of 500 μg/mL. Among these compounds, the mortalities of compounds **9m**, **9p**, and **9q** were 80.65%, 80.56%, and 80.78%, respectively, which were similar to that of the control Fenpyroximate. When the dosage was reduced to 100 μg/mL, compound **9q** was still active against *T. cinnabarinus* with inhibitory value of 70.89%. Besides acaricidal activity, most of the aimed compounds demonstrated excellent insecticidal activities against *Oriental armyworm* at a concentration of 500 μg/mL (Table 2), for instance, compounds **9a**, **9c**, **9d**, **9e**, **9f**, **9g**, **9h**, **9i**, **9j**, **9k**, **9l**, **9m**, **9n**, **9o**, **9q**, **9r**, **9s**, **9t**, and **9u** all had over 90.00% inhibition rates, which were comparable to that of the control Abamectin (100.00%). As shown in Table 2, some title compounds indicated good insecticidal activities against *A. medicaginis* at a concentration of 500 μg/mL, for example, compounds **9l**, **9m**, **9p**, **9q**, **9r**, **9s**, **9t**, and **9u** all possessed 100.00% inhibition rates, which were equally to that of the control Abamectin. Interestingly, some designed compounds showed potent insecticidal activity against *N. lugens* and beyond good insecticidal activities against *A. medicaginis*. Among these compounds, compounds **9l**, **9m**, **9o**, **9p**, **9q**, **9r**, **9s**, and **9u** had a >80.00% inhibition rate at 500 μg/mL. Based on the structure-activity data, we found that when the mercapto group is at the 4-position and R_1_ is Me, the substituent R_2_ at the 4-position of the phenyl ring was methoxy (**9l**), methyl (**9m**), fluoro (**9q**) or chloro group (**9r**) or the substituent R_2_ at the 3-position of phenyl ring was fluoro atom (**9p**), it was advantageous to increase the biological activity spectrum.

#### 2.2.2. Anticancer Activities

The inhibitory activity of the target compounds **9a**–**9w** against human pancreatic carcinoma cells (Panc-1), human hepatoma cells (HepG2), and human gastric cancer cells (SGC-7901) were screened and evaluated in vitro by the MTT method using sorafenib and 5-fluorouracil as positive controls, respectively. Their IC_50_ values were presented in Table 3. As can be seen, some title compounds showed more potent antiproliferative activity on HepG2 cells than Panc-1 and SGC-7901 cells. Among these compounds, compounds **9b**, **9g**, **9l**, **9p**, **9q**, **9r**, **9s**, **9t**, **9u**, and **9v** indicated good antiproliferative activities against HepG2 cells with IC_50_ values of 1.53–17.27 μM, better than that of the control 5-fluorouracil (IC_50_ = 35.67 μM). Especially, the inhibitory effect of compound **9r**, against HepG2 cell, was 23-fold as strong as that of 5-fluorouracil. Based on the structure-antitumor activity data, we found that when the mercapto group is at the 4-position and R_1_ is Me, the 3-fluoro substituted compound **9p** and 4-chloro substituted compound **9r** exhibited more potent inhibitory activity against HepG2 cell.

All the above results implied that good biological activities can be achieved through introducing substituted pyridyl moiety into pyrazole oxime unit. To obtain more active derivatives, further studies on these compounds are well under way.

## 3. Experimental Section

### 3.1. Chemistry

#### 3.1.1. General Procedures

All reagents are commercially available and used without further purification except as indicated. The melting points were determined on an X-4 binocular microscope melting point apparatus (Beijing Tech Instrument Co., Beijing, China) and are uncorrected. ^1^H NMR and ^13^C NMR spectra were measured on a Bruker AV400 spectrometer (400 MHz, ^1^H; 100 MHz, ^13^C, Bruker, Billerica, MA, USA) in CDCl_3_ with TMS as an internal standard. Elemental analyses were determined on a Yanaco CHN Corder MT-3 elemental analyzer (Yanaco, Kyoto, Japan). The intermediate 1-methyl-3-methyl-5-chloro-1*H*-pyrazole-4-carbaldehyde (**6**) was prepared according to the reported procedures [25].

#### 3.1.2. Synthesis of Compound **2**

To a solution of 2 (or 4)-mercaptobenzoic acid (3.1 g, 20 mmol), compound **1** (3.3 g, 22 mmol) and anhydrous ethanol (100 mL) was added potassium hydroxide (2.2 g, 40 mmol) at room temperature. The resulting mixture was heated to reflux for 10 h. Then it was cooled to room temperature, allowed to settle for 2 h, and the precipitates were collected by filtration. To the solid was added 100 mL of water, followed by adding 5% hydrochloric acid to adjust the pH to 2–3. The precipitates were filtered off to obtain compound **2**. Compound **2a**: white solid; yield 65%; m.p. 218–219 °C. ^1^H NMR (DMSO-*d*_6_): δ 13.11 (s, 1H, COOH); 8.47 (d, *J* = 2.4 Hz, 1H, Py-H); 7.22–7.92 (m, 6H, Py-H and Ar-H); 4.27 (s, 2H, CH_2_). Compound **2b**: white solid; yield 70%; m.p. 180–182 °C. ^1^H NMR (DMSO-*d*_6_): δ 12.95 (s, 1H, COOH); 8.43 (d, *J* = 2.4 Hz, 1H, Py-H); 7.43–7.90 (m, 6H, Py-H and Ar-H), 4.40 (s, 2H, CH_2_).

#### 3.1.3. Synthesis of Compound **3**

To a solution of compound **2** (5.6 g, 20 mmol) in anhydrous methanol (60 mL) was added concentrated sulfuric acid (2 mL) at room temperature. The resulting mixture was heated to reflux for 8 h and cooled to room temperature, allowed to settle for 1 h, and the precipitates were obtained by filtration to give compound **3**. Compound **3a**: white solid; yield 75%; m.p. 79–80 °C. ^1^H NMR (CDCl_3_): δ 8.16 (d, *J* = 2.4 Hz, 1H, Py-H); 7.97 (d, *J* = 7.6 Hz, 1H, Ar-H); 7.66 (dd, *J*_1_ = 2.4 Hz, *J*_2_ = 8.4 Hz, 1H, Py-H); 6.70–7.46 (m, 4H, Py-H and Ar-H); 4.09 (s, 2H, CH_2_); 3.93 (s, 3H, OCH_3_). Compound **3b**: white solid; yield 83%; m.p. 101–102 °C. ^1^H NMR (CDCl_3_): δ 8.24 (d, *J* = 2.0 Hz, 1H, Py-H); 7.85 (d, *J* = 8.4 Hz, 1H, Ar-H); 7.54 (dd, *J*_1_ = 2.0 Hz, *J*_2_ = 8.0 Hz, 1H, Py-H); 7.18–7.21 (m, 3H, Py-H and Ar-H); 4.07 (s, 2H, CH_2_), 3.83 (s, 3H, OCH_3_).

#### 3.1.4. Synthesis of Compound **4**

A solution of intermediate **3** (5.9 g, 20 mmol) in anhydrous tetrahydrofuran (60 mL) was cooled in an ice-water bath followed by adding lithium aluminum hydride (1.1 g, 40 mmol) in portions. Then the resulting mixture was stirred at room temperature for 2 h. The reaction mixture was poured into water (80 mL). The precipitates were filtered off, and the filtrate was continuously extracted with ethyl acetate (3 × 50 mL). The combined organic layer was washed by water and brine, dried over anhydrous Na_2_SO_4_, and concentrated in a rotatory evaporator to afford compound **4**. Compound **4a**: white solid; yield 63%; m.p. 80–81 °C. ^1^H NMR (CDCl_3_): δ 8.14 (d, *J* = 2.0 Hz, 1H, Py-H); 7.50 (dd, *J*_1_ = 2.4 Hz, *J*_2_ = 8.0 Hz, 1H, Py-H); 7.21–7.43 (m, 5H, Py-H and Ar-H); 4.70 (s, 2H, CH_2_); 4.02 (s, 2H, CH_2_). Anal. Calcd. for C_13_H_12_ClNOS: C 58.75; H 4.55; N 5.27. Found: C 58.88; H 4.63; N 5.16. Compound **4b**: white solid; yield 71%; m.p. 73–74 °C. ^1^H NMR (CDCl_3_): δ 8.12 (d, *J* = 2.4 Hz, 1H, Py-H); 7.56 (dd, *J*_1_ = 2.8 Hz, *J*_2_ = 8.0 Hz, 1H, Py-H); 7.22–7.27 (m, 5H, Py-H and Ar-H); 4.66 (s, 2H, CH_2_); 4.01 (s, 2H, CH_2_). Anal. Calcd. for C_13_H_12_ClNOS: C 58.75; H 4.55; N 5.27. Found: C 58.63; H 4.66; N 5.39.

#### 3.1.5. Synthesis of Compound **5**

A solution of intermediate **4** (5.3 g, 20 mmol) in dichloromethane (30 mL) was cooled in ice-water bath followed by adding dropwise a mixture of thionyl chloride (8.9 g, 40 mmol) and dichloromethane (20 mL). The reaction mixture was stirred at room temperature for 3 h. The reaction mixture was quenched by trash ice, then the organic phase was separated, washed by water and saturated NaCl solution, dried over anhydrous Na_2_SO_4_, and evaporated in vacuo to produce compound **5**. Compound **5a**: yellow oil; yield 73%. ^1^H NMR (CDCl_3_): δ 8.15 (d, *J* = 2.4 Hz, 1H, Py-H); 7.22–7.53 (m, 6H, Py-H and Ar-H); 4.72 (s, 2H, CH_2_); 4.06 (s, 2H, CH_2_). Anal. Calcd. for C_13_H_11_Cl_2_NS: C 54.94; H 3.90; N 4.93. Found: C 55.07; H 3.79; N 4.81. Compound **5b**: white solid; yield 80%; m.p. 70–71 °C. ^1^H NMR (CDCl_3_): δ 8.21 (d, *J* = 2.8 Hz, 1H, Py-H); 7.25–7.58 (m, 6H, Py-H and Ar-H); 4.54 (s, 2H, CH_2_); 4.04 (s, 2H, CH_2_). Anal. Calcd. for C_13_H_11_Cl_2_NS: C 54.94; H 3.90; N 4.93. Found: C 54.81; H 3.99; N 5.05.

#### 3.1.6. General Procedure for the Preparation of **7a**–**7w**

To a solution of substituted phenol (75 mmol) in *N*,*N*-dimethylformamide (60 mL) was added potassium hydroxide (100 mmol) at room temperature. The reaction mixture was heated to 40 °C for 6 h. Then to the above mixture was added compound **6** (50 mmol) in portions, and the resulting mixture was heated to 100 °C for 12–20 h. The reaction solution was poured into water (100 mL) and was continuously extracted with ethyl acetate (3 × 50 mL). The combined organic layer was washed by water and brine, dried over anhydrous Na_2_SO_4_, and concentrated in rotatory evaporator to give compounds **7a**–**7w**, with yields ranging from 58% to 80% [26].

#### 3.1.7. General Procedure for the Preparation of **8a**–**8w**

To a well stirred solution of hydroxylamine hydrochloride (45 mmol) and potassium hydroxide (45 mmol) in methanol (60 mL) was added compound **7** (30 mmol) at room temperature. The reaction mixture was heated to reflux for 8–15 h and cooled to room temperature. The reaction solution was poured into water (100 mL). The resulting precipitate was collected by filtration and washed with water to afford compounds **8a**–**8w**, with yields ranging from 56% to 77% [26].

#### 3.1.8. General Procedure for the Preparation of **9a**–**9w**

To a well stirred solution of intermediate **5** (6 mmol), compound **8** (5 mmol), and powdered potassium carbonate (12 mmol) in anhydrous acetonitrile (25 mL) was added Cs_2_CO_3_ (1 mmol) at room temperature, the resulting mixture was heated to reflux for 8–17 h. After cooled to room temperature, the reaction mixture was filtered. After the solvent had been removed under reduced pressure, the residue was admixed with water (80 mL) and extracted with ethyl acetate (3 × 50 mL).

The combined organic layer was dried over anhydrous Na_2_SO_4_. The solvent was evaporated in vacuo, and the residue was then subjected to column chromatography using a mixture of petroleum ether and ethyl acetate as an eluent to produce the title compounds **9a**–**9w**, with yields ranging from 53% to 75%. All 23 pyrazole oxime derivatives **9a**–**9w** were novel and the physical and spectral data for these compounds are listed below. ^1^H NMR and ^13^C NMR spectra are provided in the Supplementary Materials.

*1-Methyl-3-methyl-5-(4-methoxyphenoxy)-1H-pyrazole-4-carbaldehyde-O-[2-(2-chloropyridin-5-yl-methylthio)phenylmethyl]-oxime* (**9a**). Yellow oil, yield 62%. ^1^H NMR (CDCl_3_): δ 8.02 (s, 1H, Py-H), 7.70 (s, 1H, CH=N), 7.38 (d, *J* = 8.0 Hz, 1H, Py-H), 7.27 (d, *J* = 6.4 Hz, 1H, Py-H), 7.09–7.18 (m, 4H, Ar-H), 6.73 (s, 4H, Ar-H), 5.05 (s, 2H, CH_2_), 3.88 (s, 2H, CH_2_), 3.68 (s, 3H, OCH_3_), 3.52 (s, 3H, N-CH_3_), 2.27 (s, 3H, CH_3_); ^13^C NMR (CDCl_3_): δ 155.8, 150.7, 150.1, 149.6, 148.5, 146.9, 141.0, 139.4, 139.1, 133.6, 132.9, 132.6, 130.6, 128.6, 127.9, 123.9, 116.4, 114.9, 99.8, 74.1, 55.7, 36.2, 34.2, 14.9. Anal. Calcd. for C_2__6_H_2__5_ClN_4_O_3_S: C 61.35; H 4.95; N 11.01. Found: C 61.48; H 4.86; N 11.13.

*1-Methyl-3-methyl-5-(4-methylphenoxy)-1H-pyrazole-4-carbaldehyde-O-[2-(2-chloropyridin-5-yl-methylthio)phenylmethyl]-oxime* (**9b**). White oil, yield 60%. ^1^H NMR (CDCl_3_): δ 8.09 (d, *J* = 2.0 Hz, 1H, Py-H), 7.78 (s, 1H, CH=N), 7.33–7.46 (m, 2H, Py-H and Ar-H), 7.16–7.26 (m, 4H, Ar-H and Py-H), 7.06 (d, *J* = 8.4 Hz, 2H, Ar-H), 6.76 (d, *J* = 8.4 Hz, 2H, Ar-H), 5.12 (s, 2H, CH_2_), 3.95 (s, 2H, CH_2_), 3.57 (s, 3H, N-CH_3_), 2.35 (s, 3H, CH_3_), 2.28 (s, 3H, CH_3_); ^13^C NMR (CDCl_3_): δ 154.7, 150.1, 149.6, 148.2, 146.8, 140.9, 139.2, 139.1, 133.6, 133.2, 132.8, 132.6, 130.6, 130.4, 128.6, 127.9, 123.9, 115.1, 100.0, 74.1, 36.2, 34.2, 20.6, 14.9 Anal. Calcd. for C_2__6_H_2__5_ClN_4_O_2_S: C 63.34; H 5.11; N 11.36. Found: C 63.22; H 5.23; N 11.47.

*1-Methyl-3-methyl-5-(2-fluorophenoxy)-1H-pyrazole-4-carbaldehyde-O-[2-(2-chloropyridin-5-yl-methylthio)phenylmethyl]-oxime* (**9c**). Yellow oil, yield 53%. ^1^H NMR (CDCl_3_): δ 8.10 (d, *J* = 2.4 Hz, 1H, Py-H), 7.79 (s, 1H, CH=N), 7.44–7.47 (m, 1H, Py-H), 7.12–7.32 (m, 6H, Ar-H and Py-H), 6.96–7.05 (m, 2H, Ar-H), 6.73–6.77 (m, 1H, Ar-H), 5.08 (s, 2H, CH_2_), 3.96 (s, 2H, CH_2_), 3.64 (s, 3H, N-CH_3_), 2.32 (s, 3H, CH_3_); ^13^C NMR (CDCl_3_): δ 151.6 (d, *J* = 247 Hz), 150.1, 149.6, 147.3, 146.9, 144.2, 140.4, 139.1, 139.0, 133.6, 132.7, 132.5, 130.5, 128.7, 127.8, 124.6, 124.0, 117.1 (d, *J* = 17 Hz), 116.7, 99.8, 74.1, 36.1, 34.2, 14.6. Anal. Calcd. for C_2__5_H_22_ClFN_4_O_2_S: C 60.42; H 4.46; N 11.27. Found: C 60.29; H 4.55; N 11.14.

*1-Methyl-3-methyl-5-(3-fluorophenoxy)-1H-pyrazole-4-carbaldehyde-O-[2-(2-chloropyridin-5-yl-methylthio)phenylmethyl]-oxime* (**9d**). Yellow oil, yield 55%. ^1^H NMR (CDCl_3_): δ 8.03 (d, *J* = 2.0 Hz, 1H, Py-H), 7.73 (s, 1H, CH=N), 7.37–7.40 (m, 1H, Py-H), 7.10–7.24 (m, 6H, Ar-H and Py-H), 6.54–6.74 (m, 3H, Ar-H), 5.02 (s, 2H, CH_2_), 3.89 (s, 2H, CH_2_), 3.52 (s, 3H, N-CH_3_), 2.28 (s, 3H, CH_3_); ^13^C NMR (CDCl_3_): δ 163.5 (d, *J* = 246 Hz), 157.5, 150.1, 149.6, 147.0, 140.5, 139.1, 139.0, 133.7, 132.6, 130.9, 130.8, 130.5, 128.7, 127.8, 124.0, 110.6 (d, *J* = 29 Hz), 113.4, 103.4 (d, *J* = 26 Hz), 100.3, 74.1, 36.1, 34.3, 14.7. Anal. Calcd. for C_2__5_H_22_ClFN_4_O_2_S: C 60.42; H 4.46; N 11.27. Found: C 60.53; H 4.34; N 11.20.

*1-Methyl-3-methyl-5-(4-fluorophenoxy)-1H-pyrazole-4-carbaldehyde-O-[2-(2-chloropyridin-5-yl-methylthio)phenylmethyl]-oxime* (**9e**). Yellow oil, yield 58%. ^1^H NMR (CDCl_3_): δ 8.12 (d, *J* = 2.4 Hz, 1H, Py-H), 7.80 (s, 1H, CH=N), 7.47–7.50 (m, 1H, Py-H), 7.20–7.33 (m, 5H, Py-H and Ar-H), 6.83–7.00 (m, 4H, Ar-H), 5.10 (s, 2H, CH_2_), 3.98 (s, 2H, CH_2_), 3.62 (s, 3H, N-CH_3_), 2.36 (s, 3H, CH_3_); ^13^C NMR (CDCl_3_): δ 158.7 (d, *J* = 241 Hz), 152.6, 150.2, 149.6, 147.8, 147.0, 141.6, 140.6, 139.1, 133.7, 132.7, 130.4, 128.7, 127.8, 124.0, 116.4 (d, *J* = 31 Hz), 74.1, 36.2, 34.2, 14.6. Anal. Calcd. for C_2__5_H_22_ClFN_4_O_2_S: C 60.42; H 4.46; N 11.27. Found: C 60.31; H 4.37; N 11.39.

*1-Methyl-3-methyl-5-(2-chlorophenoxy)-1H-pyrazole-4-carbaldehyde-O-[2-(2-chloropyridin-5-yl-methylthio)phenylmethyl]-oxime* (**9f**). Yellow oil, yield 54%. ^1^H NMR (CDCl_3_): δ 8.10 (d, *J* = 2.4 Hz, 1H, Py-H), 7.77 (s, 1H, CH=N), 7.41–7.47 (m, 2H, Py-H and Ar-H), 7.17–7.31 (m, 5H, Ar-H and Py-H), 7.10–7.14 (m, 1H, Ar-H), 7.00–7.04 (m, 1H, Ar-H), 6.65–6.68 (m, 1H, Ar-H), 5.08 (s, 2H, CH_2_), 3.96 (s, 2H, CH_2_), 3.62 (s, 3H, N-CH_3_), 2.34 (s, 3H, CH_3_); ^13^C NMR (CDCl_3_): δ 152.1, 150.1, 149.6, 147.2, 147.0, 140.4, 139.1, 133.6, 132.7, 132.5, 130.9, 130.5, 128.7, 128.0, 127.8, 124.5, 124.0, 122.7, 115.5, 100.1, 74.2, 36.1, 34.2, 14.6. Anal. Calcd. for C_2__5_H_22_Cl_2_N_4_O_2_S: C 58.48; H 4.32; N 10.91. Found: C 58.60; H 4.21; N 10.83.

*1-Methyl-3-methyl-5-(4-chlorophenoxy)-1H-pyrazole-4-carbaldehyde-O-[2-(2-chloropyridin-5-yl-methylthio)phenylmethyl]-oxime* (**9g**). White solid, yield 60%, m.p. 75–77 °C. ^1^H NMR (CDCl_3_): δ 8.09 (d, *J* = 2.4 Hz, 1H, Py-H), 7.78 (s, 1H, CH=N), 7.45–7.48 (m, 1H, Py-H), 7.17–7.29 (m, 7H, Ar-H and Py-H), 6.81 (d, *J* = 8.8 Hz, 2H, Ar-H), 5.08 (s, 2H, CH_2_), 3.96 (s, 2H, CH_2_), 3.59 (s, 3H, N-CH_3_), 2.34 (s, 3H, CH_3_); ^13^C NMR (CDCl_3_): δ 155.2, 150.1, 149.6, 147.1, 147.0, 140.5, 139.1, 139.0, 133.6, 132.6, 132.5, 130.5, 129.9, 128.7, 127.8, 124.0, 116.6, 100.1, 74.1, 36.1, 34.2, 14.6. Anal. Calcd. for C_2__5_H_22_Cl_2_N_4_O_2_S: C 58.48; H 4.32; N 10.91. Found: C 58.35; H 4.42; N 11.03.

*1-Methyl-3-methyl-5-(4-bromophenoxy)-1H-pyrazole-4-carbaldehyde-O-[2-(2-chloropyridin-5-yl-methylthio)phenylmethyl]-oxime* (**9h**). Yellow oil, yield 59%. ^1^H NMR (CDCl_3_): δ 8.09 (d, *J* = 2.0 Hz, 1H, Py-H), 7.78 (s, 1H, CH=N), 7.45–7.48 (m, 1H, Py-H), 7.38 (d, *J* = 8.8 Hz, 2H, Ar-H), 7.18–7.27 (m, 5H, Ar-H and Py-H), 6.76 (d, *J* = 8.8 Hz, 2H, Ar-H), 5.08 (s, 2H, CH_2_), 3.96 (s, 2H, CH_2_), 3.59 (s, 3H, N-CH_3_), 2.34 (s, 3H, CH_3_); ^13^C NMR (CDCl_3_): δ 155.7, 150.1, 149.6, 147.0, 140.5, 139.1, 133.6, 132.8, 132.6, 132.5, 130.5, 128.7, 127.8, 124.0, 117.1, 116.1, 100.2, 74.2, 36.1, 34.2, 14.5. Anal. Calcd. for C_2__5_H_22_BrClN_4_O_2_S: C 53.82; H 3.97; N 10.04. Found: C 53.69; H 4.08; N 10.14.

*1-Methyl-3-methyl-5-(4-iodophenoxy)-1H-pyrazole-4-carbaldehyde-O-[2-(2-chloropyridin-5-yl-methylthio)phenylmethyl]-oxime* (**9i**). Yellow oil, yield 60%. ^1^H NMR (CDCl_3_): δ 8.02 (d, *J* = 2.4 Hz, 1H, Py-H), 7.71 (s, 1H, CH=N), 7.49 (d, *J* = 8.8 Hz, 2H, Ar-H), 7.38–7.41 (m, 1H, Py-H), 7.11–7.19 (m, 5H, Py-H and Ar-H), 6.57 (d, *J* = 8.8 Hz, 2H, Ar-H), 5.00 (s, 2H, CH_2_), 3.89 (s, 2H, CH_2_), 3.51 (s, 3H, N-CH_3_), 2.26 (s, 3H, CH_3_); ^13^C NMR (CDCl_3_): δ 155.6, 149.1, 148.6, 146.0, 139.4, 138.1, 138.0, 137.8, 132.6, 131.7, 131.5, 129.4, 127.7, 126.8, 123.0, 116.6, 99.2, 85.4, 73.2, 35.2, 33.2, 13.5. Anal. Calcd. for C_2__5_H_22_ClIN_4_O_2_S: C 49.64; H 3.67; N 9.26. Found: C 49.78; H 3.56; N 9.38.

*1-Methyl-3-methyl-5-(2,4-difluorophenoxy)-1H-pyrazole-4-carbaldehyde-O-[2-(2-chloropyridin-5-yl-methylthio)phenylmethyl]-oxime* (**9j**). Yellow oil, yield 56%. ^1^H NMR (CDCl_3_): δ 8.10 (d, *J* = 2.0 Hz, 1H, Py-H), 7.78 (s, 1H, CH=N), 7.46–7.49 (m, 1H, Py-H), 7.19–7.30 (m, 5H, Ar-H and Py-H), 6.88–6.93 (m, 1H, Ar-H), 6.70–6.75 (m, 2H, Ar-H), 5.06 (s, 2H, CH_2_), 3.97 (s, 2H, CH_2_), 3.65 (s, 3H, N-CH_3_), 2.32 (s, 3H, CH_3_); ^13^C NMR (CDCl_3_): δ 148.6 (d, *J* = 332 Hz), 148.3 (d, *J* = 308 Hz), 141.2, 140.2, 139.1, 138.9, 133.6, 132.5, 131.1, 130.3, 128.7, 128.5, 127.8, 124.0, 117.5, 111.0 (d, *J* = 23 Hz), 105.6 (d, *J* = 22 Hz), 105.4 (d, *J* = 22 Hz), 99.5, 74.1, 36.1, 34.2, 14.4. Anal. Calcd. for C_2__5_H_21_ClF_2_N_4_O_2_S: C 58.31; H 4.11; N 10.88. Found: C 58.45; H 4.04; N 10.76.

*1-Methyl-3-methyl-5-(2,4-dichlorophenoxy)-1H-pyrazole-4-carbaldehyde-O-[2-(2-chloropyridin-5-yl-methylthio)phenylmethyl]-oxime* (**9k**). Yellow oil, yield 57%.^1^H NMR (CDCl_3_): δ 8.10 (d, *J* = 2.4 Hz, 1H, Py-H), 7.78 (s, 1H, CH=N), 7.41–7.48 (m, 2H, Ar-H and Py-H), 7.05–7.26 (m, 6H, Ar-H and Py-H), 6.59 (d, *J* = 8.8 Hz, 1H, Ar-H), 5.05 (s, 2H, CH_2_), 3.97 (s, 2H, CH_2_), 3.63 (s, 3H, N-CH_3_), 2.32 (s, 3H, CH_3_); ^13^C NMR (CDCl_3_): δ 150.8, 150.1, 149.6, 147.1, 146.5, 140.1, 139.1, 138.9, 133.6, 132.5, 130.5, 130.3, 129.0, 128.7, 127.9, 127.8, 124.0, 123.6, 116.3, 100.1, 74.2, 36.1, 34.3, 14.3. Anal. Calcd. for C_2__5_H_21_Cl_3_N_4_O_2_S: C 54.80; H 3.86; N 10.23. Found: C 54.93; H 3.76; N 10.12.

*1-Methyl-3-methyl-5-(4-methoxyphenoxy)-1H-pyrazole-4-carbaldehyde-O-[4-(2-chloropyridin-5-yl-methylthio)phenylmethyl]-oxime* (**9l**). Yellow oil, yield 75%. ^1^H NMR (CDCl_3_): δ 8.19 (d, *J* = 2.4 Hz, 1H, Py-H), 7.78 (s, 1H, CH=N), 7.52–7.54 (m, 1H, Py-H), 7.19–7.27 (m, 5H, Py-H and Ar-H), 6.82 (s, 4H, Ar-H), 4.95 (s, 2H, CH_2_), 4.02 (s, 2H, CH_2_), 3.78 (s, 3H, OCH_3_), 3.59 (s, 3H, N-CH_3_), 2.34 (s, 3H, CH_3_); ^13^C NMR (CDCl_3_): δ 155.7, 150.6, 150.2, 149.6, 148.4, 146.8, 141.0, 139.0, 137.0, 133.8, 132.6, 131.5, 130.8, 129.3, 124.1, 116.3, 114.9, 99.8, 75.3, 55.7, 35.8, 34.2, 14.8. Anal. Calcd. for C_2__6_H_2__5_ClN_4_O_3_S: C 61.35; H 4.95; N 11.01. Found: C 61.23; H 4.85; N 11.10.

*1-Methyl-3-methyl-5-(4-methylphenoxy)-1H-pyrazole-4-carbaldehyde-O-[4-(2-chloropyridin-5-yl-methylthio)phenylmethyl]-oxime* (**9m**). Yellow oil, yield 73%. ^1^H NMR (CDCl_3_): δ 8.21 (d, *J* = 2.4 Hz, 1H, Py-H), 7.81 (s, 1H, CH=N), 7.53–7.56 (m, 1H, Py-H), 7.21–7.24 (m, 5H, Py-H and Ar-H), 7.11 (d, *J* = 8.4 Hz, 2H, Ar-H), 6.78 (d, *J* = 8.4 Hz, 2H, Ar-H), 4.97 (s, 2H, CH_2_), 4.03 (s, 2H, CH_2_), 3.60 (s, 3H, N-CH_3_), 2.37 (s, 3H, CH_3_), 2.33 (s, 3H, CH_3_); ^13^C NMR (CDCl_3_): δ 154.7, 150.2, 149.6, 148.1, 146.8, 141.0, 139.0, 137.1, 133.8, 133.2, 132.6, 130.9, 130.4, 129.2, 124.1, 115.1, 100.1, 75.4, 35.9, 34.2, 20.6, 14.9. Anal. Calcd. for C_2__6_H_2__5_ClN_4_O_2_S: C 63.34; H 5.11; N 11.36. Found: C 63.26; H 5.20; N 11.24.

*1-Methyl-3-methyl-5-(4-trifluoromethoxyphenoxy)-1H-pyrazole-4-carbaldehyde-O-[4-(2-chloropyridin-5-yl-methylthio)phenylmethyl]-oxime* (**9n**). Yellow oil, yield 68%. ^1^H NMR (CDCl_3_): δ 8.19 (s, 1H, Py-H), 7.80 (s, 1H, CH=N), 7.55 (d, *J* = 6.8 Hz, 1H, Py-H), 7.15–7.23 (m, 7H, Py-H and Ar-H), 6.88 (d, *J* = 8.8 Hz, 2H, Ar-H), 4.90 (s, 2H, CH_2_), 4.02 (s, 2H, CH_2_), 3.61 (s, 3H, N-CH_3_), 2.34 (s, 3H, CH_3_); ^13^C NMR (CDCl_3_): δ 154.8, 150.2, 149.5, 147.2, 146.9, 144.9, 140.2, 139.1, 136.7, 134.0, 132.6, 130.8, 129.2, 124.2, 122.9, 116.5, 100.4, 75.5, 35.7, 34.3, 14.4. Anal. Calcd. for C_2__6_H_22_ClF_3_N_4_O_3_S: C 55.47; H 3.94; N 9.95. Found: C 55.60; H 3.82; N 9.84.

*1-Methyl-3-methyl-5-(2-fluorophenoxy)-1H-pyrazole-4-carbaldehyde-O-[4-(2-chloropyridin-5-yl-methylthio)phenylmethyl]-oxime* (**9o**). Yellow solid, yield 61%, m.p. 58–60 °C. ^1^H NMR (CDCl_3_): δ 8.19 (d, *J* = 2.4 Hz, 1H, Py-H), 7.79 (s, 1H, CH=N), 7.52–7.55 (m, 1H, Py-H), 7.14–7.23 (m, 6H, Py-H and Ar-H), 6.98–7.08 (m, 2H, Ar-H), 6.72–6.77 (m, 1H, Ar-H), 4.91 (s, 2H, CH_2_), 4.02 (s, 2H, CH_2_), 3.65 (s, 3H, N-CH_3_), 2.33 (s, 3H, CH_3_); ^13^C NMR (CDCl_3_): δ 152.0 (d, *J* = 247 Hz), 150.2, 149.6, 147.3, 147.0, 144.3, 140.4, 139.0, 137.0, 133.9, 132.6, 130.9, 129.2, 124.5, 124.1, 117.1 (d, *J* = 14 Hz), 116.8, 99.9, 75.4, 35.9, 34.2, 14.5. Anal. Calcd. for C_2__5_H_22_ClFN_4_O_2_S: C 60.42; H 4.46; N 11.27. Found: C 60.51; H 4.34; N 11.40.

*1-Methyl-3-methyl-5-(3-fluorophenoxy)-1H-pyrazole-4-carbaldehyde-O-[4-(2-chloropyridin-5-yl-methylthio)phenylmethyl]-oxime* (**9p**). Yellow oil, yield 64%. ^1^H NMR (CDCl_3_): δ 8.19 (d, *J* = 2.4 Hz, 1H, Py-H), 7.81 (s, 1H, CH=N), 7.53–7.55 (m, 1H, Py-H), 7.16–7.23 (m, 6H, Py-H and Ar-H), 6.60–6.83 (m, 3H, Ar-H), 4.93 (s, 2H, CH_2_), 4.02 (s, 2H, CH_2_), 3.60 (s, 3H, N-CH_3_), 2.35 (s, 3H, CH_3_); ^13^C NMR (CDCl_3_): δ 163.6 (d, *J* = 248 Hz), 157.6, 150.6, 150.2, 149.6, 147.0, 141.9, 140.9, 140.5, 139.0, 136.9, 133.9, 132.6, 130.8, 129.2, 128.9, 124.1, 110.6 (d, *J* = 22 Hz), 103.5 (d, *J* = 21 Hz), 100.3, 75.4, 35.8, 34.2, 14.6. Anal. Calcd. for C_2__5_H_22_ClFN_4_O_2_S: C 60.42; H 4.46; N 11.27. Found: C 60.33; H 4.53; N 11.17.

*1-Methyl-3-methyl-5-(4-fluorophenoxy)-1H-pyrazole-4-carbaldehyde-O-[4-(2-chloropyridin-5-yl-methylthio)phenylmethyl]-oxime* (**9q**). White solid, yield 66%, m.p. 39–41 °C. ^1^H NMR (CDCl_3_): δ 8.19 (d, *J* = 2.4 Hz, 1H, Py-H), 7.79 (s, 1H, CH=N), 7.53–7.56 (m, 1H, Py-H), 7.16–7.23 (m, 5H, Py-H and Ar-H), 6.82~7.00 (m, 4H, Ar-H), 4.93 (s, 2H, CH_2_), 4.02 (s, 2H, CH_2_), 3.60 (s, 3H, N-CH_3_), 2.34 (s, 3H, CH_3_); ^13^C NMR (CDCl_3_): δ 158.6 (d, *J* = 241 Hz), 152.6, 150.2, 149.6, 147.7, 147.0, 140.7, 139.1, 136.9, 133.9, 132.6, 130.8, 129.2, 124.1, 116.6, 116.4 (d, *J* = 14 Hz), 100.0, 75.4, 35.8, 34.3, 14.6. Anal. Calcd. for C_2__5_H_22_ClFN_4_O_2_S: C 60.42; H 4.46; N 11.27. Found: C 60.36; H 4.38; N 11.38.

*1-Methyl-3-methyl-5-(4-chlorophenoxy)-1H-pyrazole-4-carbaldehyde-O-[4-(2-chloropyridin-5-yl-methylthio)phenylmethyl]-oxime* (**9r**). Yellow oil, yield 70%. ^1^H NMR (CDCl_3_): δ 8.19 (d, *J* = 2.0 Hz, 1H, Py-H), 7.79 (s, 1H, CH=N), 7.53–7.56 (m, 1H, Py-H), 7.21–7.26 (m, 5H, Py-H and Ar-H), 7.15 (d, *J* = 8.4 Hz, 2H, Ar-H), 6.81 (d, *J* = 9.2 Hz, 2H, Ar-H), 4.92 (s, 2H, CH_2_), 4.02 (s, 2H, CH_2_), 3.60 (s, 3H, N-CH_3_), 2.33 (s, 3H, CH_3_); ^13^C NMR (CDCl_3_): δ 155.1, 150.2, 149.5, 147.2, 147.0, 140.4, 139.1, 136.8, 133.9, 132.6, 130.8, 130.0, 129.2, 128.8, 124.1, 116.7, 100.3, 75.5, 35.8, 34.3, 14.4. Anal. Calcd. for C_2__5_H_22_Cl_2_N_4_O_2_S: C 58.48; H 4.32; N 10.91. Found: C 58.61; H 4.20; N 10.82.

*1-Methyl-3-methyl-5-(4-bromophenoxy)-1H-pyrazole-4-carbaldehyde-O-[4-(2-chloropyridin-5-yl-methylthio)phenylmethyl]-oxime* (**9s**). Yellow oil, yield 68%. ^1^H NMR (CDCl_3_): δ 8.19 (d, *J* = 2.0 Hz, 1H, Py-H), 7.79 (s, 1H, CH=N), 7.54–7.56 (m, 1H, Py-H), 7.39 (d, *J* = 8.8 Hz, 2H, Ar-H), 7.14–7.23 (m, 5H, Py-H and Ar-H), 6.76 (d, *J* = 8.8 Hz, 2H, Ar-H), 4.92 (s, 2H, CH_2_), 4.03 (s, 2H, CH_2_), 3.60 (s, 3H, N-CH_3_), 2.34 (s, 3H, CH_3_); ^13^C NMR (CDCl_3_): δ 155.7, 150.2, 149.5, 147.0, 146.9, 140.5, 139.1, 136.9, 133.9, 132.8, 132.5, 130.8, 129.2, 124.1, 117.0, 116.1, 100.2, 75.4, 35.7, 34.2, 14.5. Anal. Calcd. for C_2__5_H_22_BrClN_4_O_2_S: C 53.82; H 3.97; N 10.04. Found: C 53.95; H 3.85; N 9.92.

*1-Methyl-3-methyl-5-(4-iodophenoxy)-1H-pyrazole-4-carbaldehyde-O-[4-(2-chloropyridin-5-yl-methylthio)phenylmethyl]-oxime* (**9t**). Yellow oil, yield 65%. ^1^H NMR (CDCl_3_): δ 8.20 (d, *J* = 2.4 Hz, 1H, Py-H), 7.79 (s, 1H, CH=N), 7.53–7.60 (m, 3H, Py-H and Ar-H), 7.14–7.24 (m, 5H, Py-H and Ar-H), 6.65 (d, *J* = 8.8 Hz, 2H, Ar-H), 4.92 (s, 2H, CH_2_), 4.03 (s, 2H, CH_2_), 3.59 (s, 3H, N-CH_3_), 2.33 (s, 3H, CH_3_); ^13^C NMR (CDCl_3_): δ 156.6, 150.2, 149.6, 147.0, 146.9, 140.5, 139.1, 138.9, 136.9, 133.9, 132.6, 130.9, 129.3, 124.1, 117.6, 100.3, 86.5, 75.5, 35.8, 34.3, 14.5. Anal. Calcd. for C_2__5_H_22_ClIN_4_O_2_S: C 49.64; H 3.67; N 9.26. Found: C 49.73; H 3.79; N 9.15.

*1-Methyl-3-methyl-5-(2,4-difluorophenoxy)-1H-pyrazole-4-carbaldehyde-O-[4-(2-chloropyridin-5-yl-methylthio)phenylmethyl]-oxime* (**9u**). Yellow oil, yield 63%. ^1^H NMR (CDCl_3_): δ 8.19 (d, *J* = 2.4 Hz, 1H, Py-H), 7.78 (s, 1H, CH=N), 7.54–7.56 (m, 1H, Py-H), 7.16–7.37 (m, 5H, Py-H and Ar-H), 6.91–6.94 (m, 1H, Ar-H), 6.71–6.74 (m, 2H, Ar-H), 4.91 (s, 2H, CH_2_) 4.02 (s, 2H, CH_2_), 3.66 (s, 3H, N-CH_3_), 2.31 (s, 3H, CH_3_); ^13^C NMR (CDCl_3_): δ 149.6, 148.9 (d, *J* = 334 Hz), 148.6 (d, *J* = 316 Hz), 147.1, 140.6, 140.3, 139.0, 137.0, 133.9, 132.6, 130.8, 129.1, 128.7, 124.2, 124.1, 117.5, 111.0 (d, *J* = 21 Hz), 105.6 (d, *J* = 21 Hz), 99.6, 75.4, 35.8, 34.2, 14.3. Anal. Calcd. for C_2__5_H_21_ClF_2_N_4_O_2_S: C 58.31; H 4.11; N 10.88. Found: C 58.18; H 4.23; N 10.99.

*1-Methyl-3-methyl-5-(2,4-dichlorophenoxy)-1H-pyrazole-4-carbaldehyde-O-[4-(2-chloropyridin-5-yl-methylthio)phenylmethyl]-oxime* (**9v**). Yellow oil, yield 66%. ^1^H NMR (CDCl_3_): δ 8.19 (s, 1H, Py-H), 7.78 (s, 1H, CH=N), 7.43–7.56 (m, 2H, Py-H and Ar-H), 7.08–7.23 (m, 6H, Py-H and Ar-H), 6.59 (d, *J* = 8.8 Hz, 1H, Ar-H), 4.90 (s, 2H, CH_2_) 4.02 (s, 2H, CH_2_), 3.63 (s, 3H, N-CH_3_), 2.32 (s, 3H, CH_3_); ^13^C NMR (CDCl_3_): δ 150.9, 150.2, 149.6, 147.2, 146.5, 140.1, 139.0, 136.9, 133.9, 132.6, 130.8, 130.5, 129.1, 129.0, 127.9, 124.1, 123.6, 116.3, 100.1, 75.4, 35.8, 34.2, 14.2. Anal. Calcd. for C_2__5_H_21_Cl_3_N_4_O_2_S: C 54.80; H 3.86; N 10.23. Found: C 54.67; H 3.97; N 10.35.

*1-(4-Methylphenyl)-3-methyl-5-(4-methylphenoxy)-1H-pyrazole-4-carbaldehyde-O-[4-(2-chloropyridin-5-yl-methylthio)phenylmethyl]-oxime* (**9w**). Yellow oil, yield 65%. ^1^H NMR (CDCl_3_): δ 8.20 (d, *J* = 2.4 Hz, 1H, Py-H), 7.81 (s, 1H, CH=N), 7.51–7.54 (m, 1H, Py-H), 7.45 (d, *J* = 8.4 Hz, 2H, Ar-H), 7.15–7.22 (m, 5H, Py-H and Ar-H), 7.14 (d, *J* = 8.4 Hz, 2H, Ar-H), 7.03 (d, *J* = 8.4 Hz, 2H, Ar-H), 6.77 (d, *J* = 8.8 Hz, 2H, Ar-H), 4.97 (s, 2H, CH_2_), 4.01 (s, 2H, CH_2_), 2.43 (s, 3H, CH_3_), 2.31 (s, 3H, CH_3_), 2.27 (s, 3H, CH_3_); ^13^C NMR (CDCl_3_): δ 154.8, 150.2, 149.6, 148.1, 147.5, 140.8, 139.0, 137.1, 135.1, 133.9, 133.1, 132.6, 130.9, 130.3, 129.7, 129.2, 128.9, 124.1, 122.1, 115.3, 101.6, 75.5, 35.9, 21.0, 20.6, 15.1. Anal. Calcd. for C_32_H_29_ClN_4_O_2_S: C 67.53; H 5.14; N 9.84. Found: C 67.66; H 5.03; N 9.95.

### 3.2. Biological Tests

#### 3.2.1. Acaricidal Activity and Insecticidal Activity Assay

All bioassays were performed on representative test organisms reared in the laboratory. The bioassay was repeated in triplicate. Assessments were made on a dead/alive basis, and mortality rates were corrected using Abbott’s formula. The acaricidal activities against *Tetranychus cinnabarinus*, and insecticidal activities against *Aphis medicaginis* and *Nilaparvata lugens* of the target compounds were tested by the spray method [26]. Under the Potter spray tower, horsebean leaves inoculated with *Tetranychus cinnabarinus* were separately treated with solutions of tested compounds. After that, the resultant horsebean leaves were kept in an observation room for normal cultivation at 24 °C~27 °C. Mortality was assessed 48 h after treatment. Each test was run three times and results were averaged. Fenpyroximate was used as the control. Activities against *Aphis medicaginis* were evaluated by the similar procedure except that the culture temperature was reduced to 20~22 °C. Abamectin was selected as the control. Inhibitions of *Nilaparvata lugens* were tested on the rice seedlings, which was inoculated with *N. lugens* first. After that, the resultant rice seedlings were kept in an observation room for normal cultivation at 24~27 °C. Mortality was assessed 48 h after treatment. All the tests were run with three duplicates and the results were averaged. Abamectin was also used as the control. The larvicidal activities of the aimed compounds against *Oriental armyworm* were evaluated by foliar application. Corn leaves were dipped into the obtained solutions for 2–3 s. After air-drying, the soaked leaves were put into a culture dish with a piece of filter paper, followed by inoculation of 10 third-instar *Oriental armyworm* larvae per dish. Covered with gauze and then kept in an observation room for normal cultivation at 24~27 °C. Mortality was assessed 48 h after treatment. The individuals who didn’t respond to the touch of writing brush were recognized as dead. Each test was run three times and results were averaged. Abamectin as the control compound was tested under the same condition.

#### 3.2.2. Anticancer Activity Assay

Panc-1 (human pancreatic carcinoma cells), HepG2 (human hepatoma cells), or SGC-7901 (human gastric cancer cells) at 10^4^ cells per well were cultured overnight in 10% FBS DMEM in 96-well flat-bottom microplates [27]. The cells were incubated in triplicate with, or without, different concentrations of each test compound for 48 h. During the last 4 h incubation, 30 μL of tetrazolium dye (MTT) solution (5 mg/mL) was added to each well. The resulting MTT-formazan crystals were dissolved in 150 μL DMSO, and absorbance was measured spectrophotometrically at 570 nm using an ELISA plate reader. The inhibition induced by each test compound at the indicated concentrations was expressed as a percentage. The concentration required for 50% inhibition (IC_50_) was calculated using the software (Graph Pad Prism, San Diego, CA, USA, Version 4.03).

## 4. Conclusions

In summary, a number of pyrazole oxime derivatives containing a substituted pyridyl subunit were synthesized. Preliminary bioassays indicated that some of the title compounds showed wonderful acaricidal activity against *T. cinnabarinus* at a concentration of 500 μg/mL, among these derivatives, compound **9q** still exhibited moderate acaricidal activity against *T. cinnabarinus* under the concentration of 100 μg/mL. Moreover, some target compounds were active against *Oriental armyworm*, *A. medicaginis*, and *N. lugen* at 500 μg/mL. Furthermore, some compounds such as **9b**, **9g**, **9l**, **9p**, **9q**, **9r**, **9s**, **9t**, **9u**, and **9v** possessed potent antiproliferative activities against HepG2 cells with IC_50_ values of 12.65, 17.27, 8.72, 7.24, 8.27, 1.53, 9.76, 9.13, 15.24, and 11.93 μM, respectively, which were better than that of 5-fluorouracil (IC_50_ = 35.67 μM). Further structural optimization and bioactivities about these pyrazole oximes are currently in progress.

## Figures and Tables

**Table 1:** Acaricidal activities of compounds **9a**–**9w** (mortality, %).

Compd.	*Tetranychus cinnabarinus*		
	500 μg/mL	100 μg/mL	20 μg/mL
**9a**	0	— ^b^	—
**9b**	0	—	—
**9c**	0	—	—
**9d**	0	—	—
**9e**	0	—	—
**9f**	0	—	—
**9g**	0	—	—
**9h**	0	—	—
**9i**	0	—	—
**9j**	0	—	—
**9k**	0	—	—
**9l**	50.39 ± 0.83 ^a^	—	—
**9m**	80.65 ± 1.32	0	—
**9n**	0	—	—
**9o**	0	—	—
**9p**	80.56 ± 1.05	0	—
**9q**	80.78 ± 0.76	70.89 ± 1.13	30.55 ± 0.35
**9r**	40.92 ± 1.68	—	—
**9s**	0	—	—
**9t**	0	—	—
**9u**	0	—	—
**9v**	0	—	—
**9w**	0	—	—
Fenpyroximate	100.00 ± 0.00	100.00 ± 0.00	100.00 ± 0.00

^a^ Each value represents the mean ± standard error of three replications; ^b^ “—” refers to “not tested”.

**Table 2:** Insecticidal activities of compounds **9a**–**9w** (mortality, %).

Compd.	*Oriental armyworm*		*Aphis medicaginis*		*Nilaparvata lugens*	
	500 μg/mL	100 μg/mL	500 μg/mL	100 μg/mL	500 μg/mL	100 μg/mL
**9a**	100.00 ± 0.00 ^a^	0	0	—	0	—
**9b**	80.23 ± 1.22	0	0	—	0	—
**9c**	100.00 ± 0.00	0	0	—	0	—
**9d**	100.00 ± 0.00	0	0	—	70.57 ± 1.26	0
**9e**	100.00 ± 0.00	20.41 ± 1.02	0	—	0	—
**9f**	100.00 ± 0.00	0	0	—	50.68 ± 1.72	—
**9g**	100.00 ± 0.00	0	0	—	60.36 ± 0.96	—
**9h**	90.36 ± 1.35	0	0	—	0	—
**9i**	100.00 ± 0.00	0	0	—	0	—
**9j**	100.00 ± 0.00	0	0	—	0	—
**9k**	100.00 ± 0.00	0	0	—	0	—
**9l**	100.00 ± 0.00	0	100.00 ± 0.00	20.88 ± 1.52	100.00 ± 0.00	0
**9m**	100.00 ± 0.00	0	100.00 ± 0.00	0	100.00 ± 0.00	0
**9n**	100.00 ± 0.00	0	0	—	0	—
**9o**	100.00 ± 0.00	0	0	—	95.54 ± 1.62	0
**9p**	60.45 ± 0.82	— ^b^	100.00 ± 0.00	0	100.00 ± 0.00	0
**9q**	90.56 ± 1.91	0	100.00 ± 0.00	0	100.00 ± 0.00	0
**9r**	100.00 ± 0.00	30.62 ± 0.87	100.00 ± 0.00	0	90.66 ± 1.08	0
**9s**	100.00 ± 0.00	0	100.00 ± 0.00	0	100.00 ± 0.00	0
**9t**	100.00 ± 0.00	40.37 ± 1.18	100.00 ± 0.00	0	60.53 ± 1.37	—
**9u**	100.00 ± 0.00	0	100.00 ± 0.00	0	80.68 ± 0.29	0
**9v**	80.33 ± 0.79	0	50.77 ± 0.42	—	0	—
**9w**	80.25 ± 1.22	0	0	—	0	—
Abamectin	100.00 ± 0.00	100.00 ± 0.00	100.00 ± 0.00	100.00 ± 0.00	100.00 ± 0.00	100.00 ± 0.00

^a^ Each value represents the mean ± standard error of three replications; ^b^ “—” refers to “not tested”.

**Table 3:** Antiproliferative activity of compounds **9a**–**9w**.

Compd.	IC_50_, μM		
	Panc-1	HepG2	SGC-7901
**9a**	>40	>40	>40
**9b**	>40	12.65 ± 0.75	>40
**9c**	>40	>40	>40
**9d**	>40	39.35 ± 0.60	>40
**9e**	>40	>40	>40
**9f**	>40	39.52 ± 0.51	>40
**9g**	>40	17.27 ± 2.02	>40
**9h**	>40	39.02 ± 0.93	>40
**9i**	>40	>40	>40
**9j**	>40	>40	>40
**9k**	>40	>40	>40
**9l**	>40	8.72 ± 1.23	>40
**9m**	>40	>40	>40
**9n**	>40	33.46 ± 2.55	>40
**9o**	>40	>40	>40
**9p**	>40	7.24 ± 0.54	>40
**9q**	>40	8.27 ± 1.47	>40
**9r**	>40	1.53 ± 0.79	>40
**9s**	>40	9.76 ± 2.32	>40
**9t**	>40	9.13 ± 1.73	>40
**9u**	>40	15.24 ± 3.05	>40
**9v**	>40	11.93 ± 2.24	>40
**9w**	>40	>40	>40
Sorafenib	11.50 ± 2.32	—	12.10 ± 2.68
5-Fluorouracil	—	35.67 ± 3.15	—

“—” refers to not tested.

## Supplementary Materials

Supplementary materials are available online: Figures S1–S46.
