# Supplementary material for: Design, Synthesis, and Biological Activities of Novel Pyrazole Oxime Compounds Containing a Substituted Pyridyl Moiety
Source: Molecules. 2017 May 25;22(6):878. doi: 10.3390/molecules22060878 (PMC6152754; doi:10.3390/molecules22060878)
Supplement: Supplementary file 1 [file molecules-22-00878-s001.pdf]

molecules

MDPI

Supplementary Materials

The ^{1}H-NMR and ^{13}C-NMR spectra of pyrazole oxime derivatives (**9a**–**9w**) were listed below:

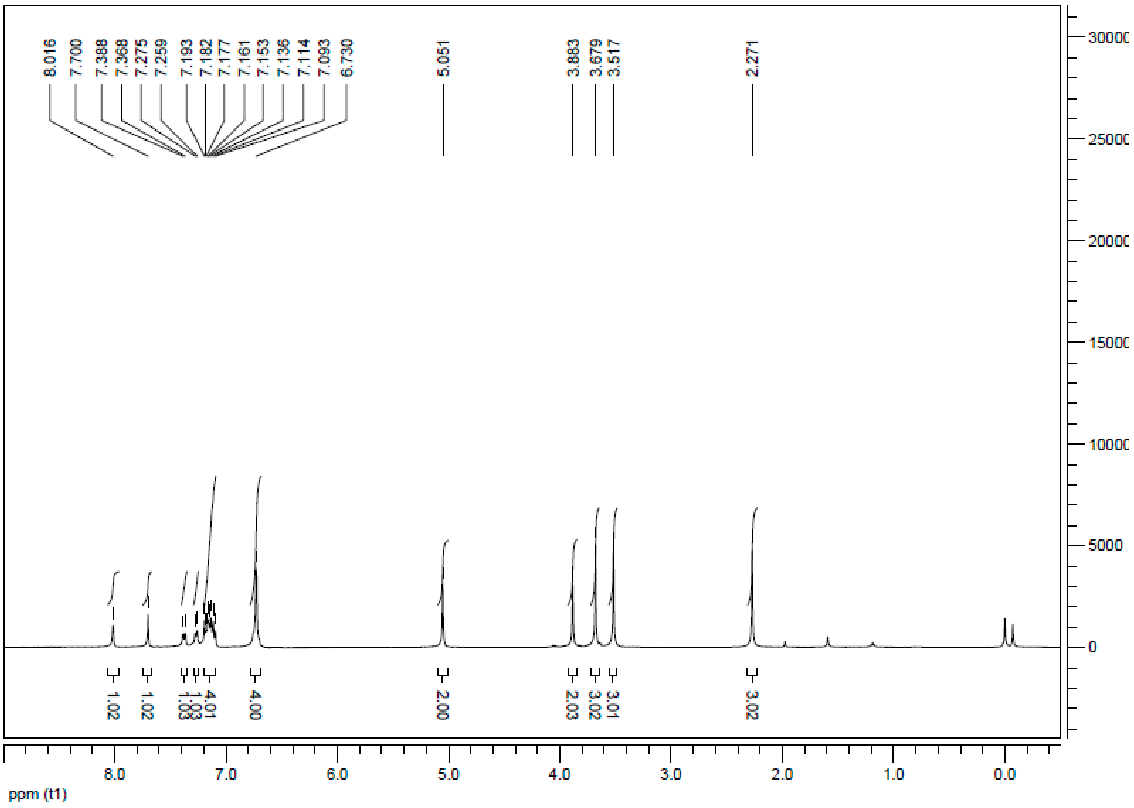

Figure S1. ^{1}H-NMR of compound **9a** (400 MHz, $CDCl_3$).

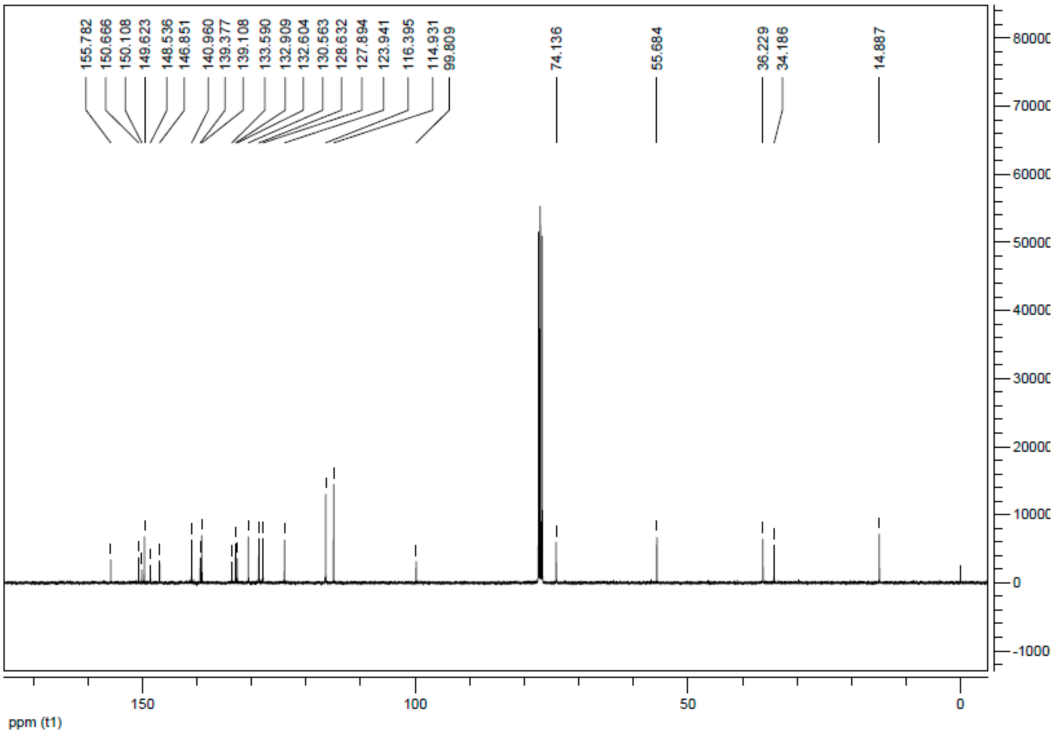

Figure S2. ^{13}C-NMR of compound **9a** (100 MHz, $CDCl_3$).

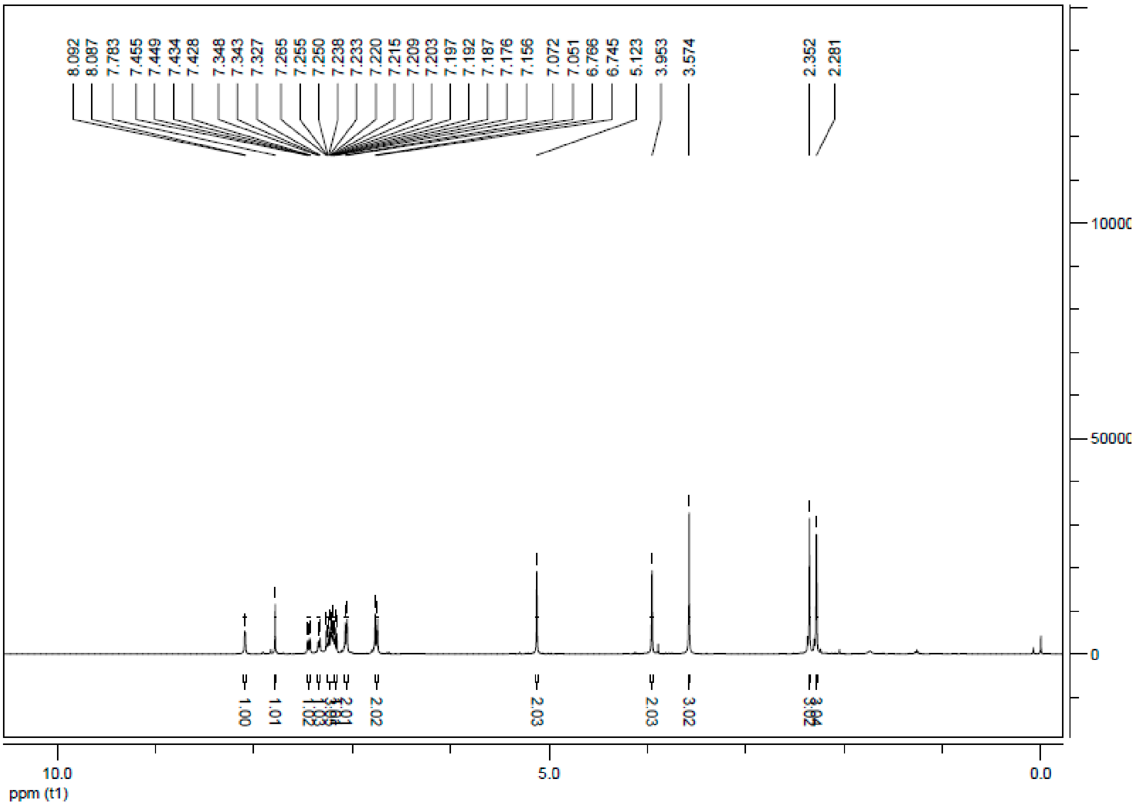

Figure S3. ^{1}H-NMR of compound **9b** (400 MHz, $CDCl_3$).

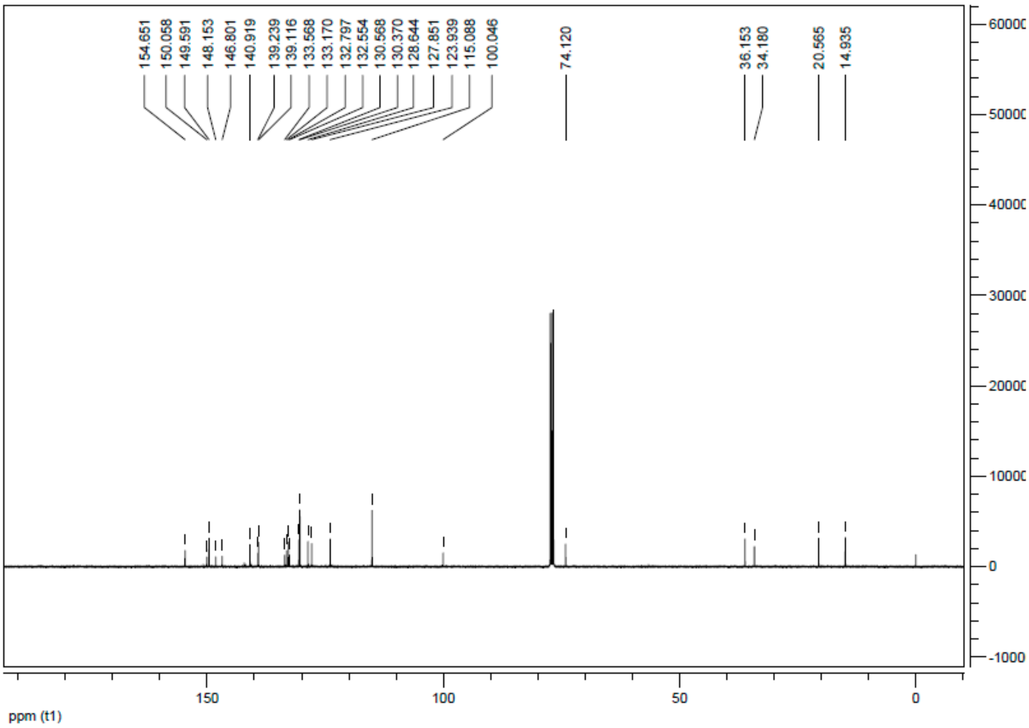

Figure S4. ^{13}C-NMR of compound **9b** (100 MHz, $CDCl_3$).

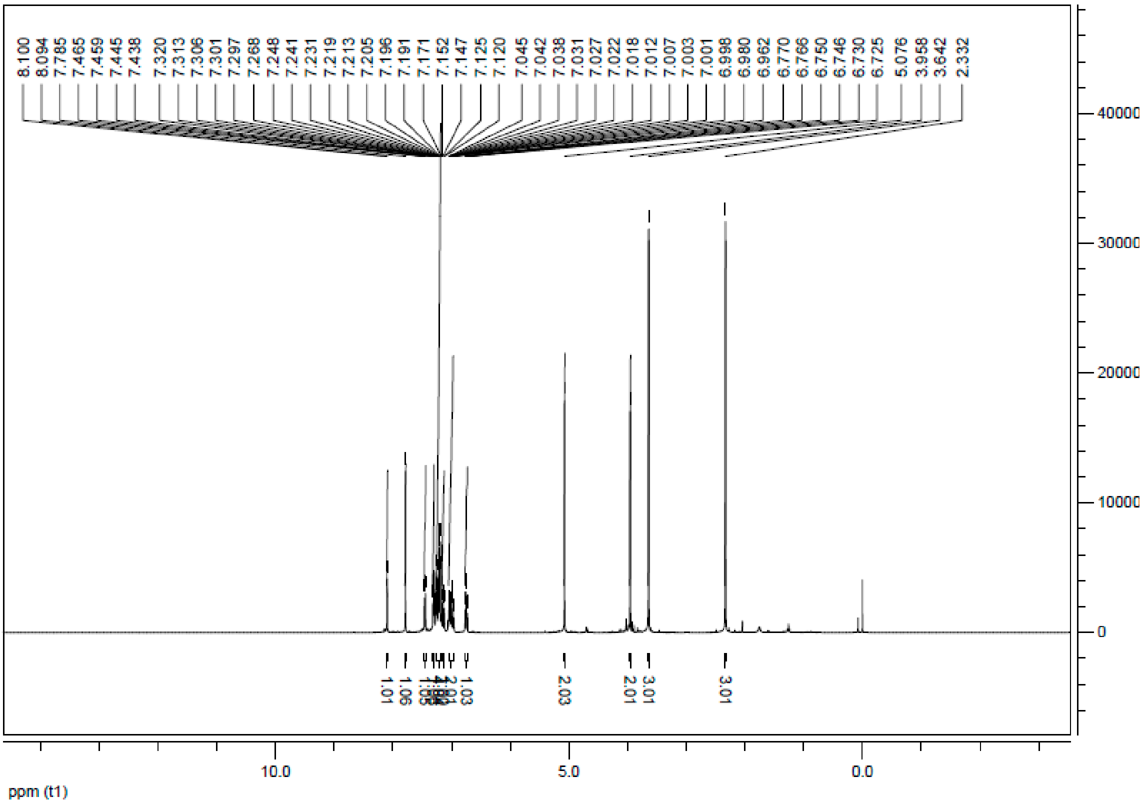

Figure S5. ^{1}H-NMR of compound **9c** (400 MHz, $CDCl_3$).

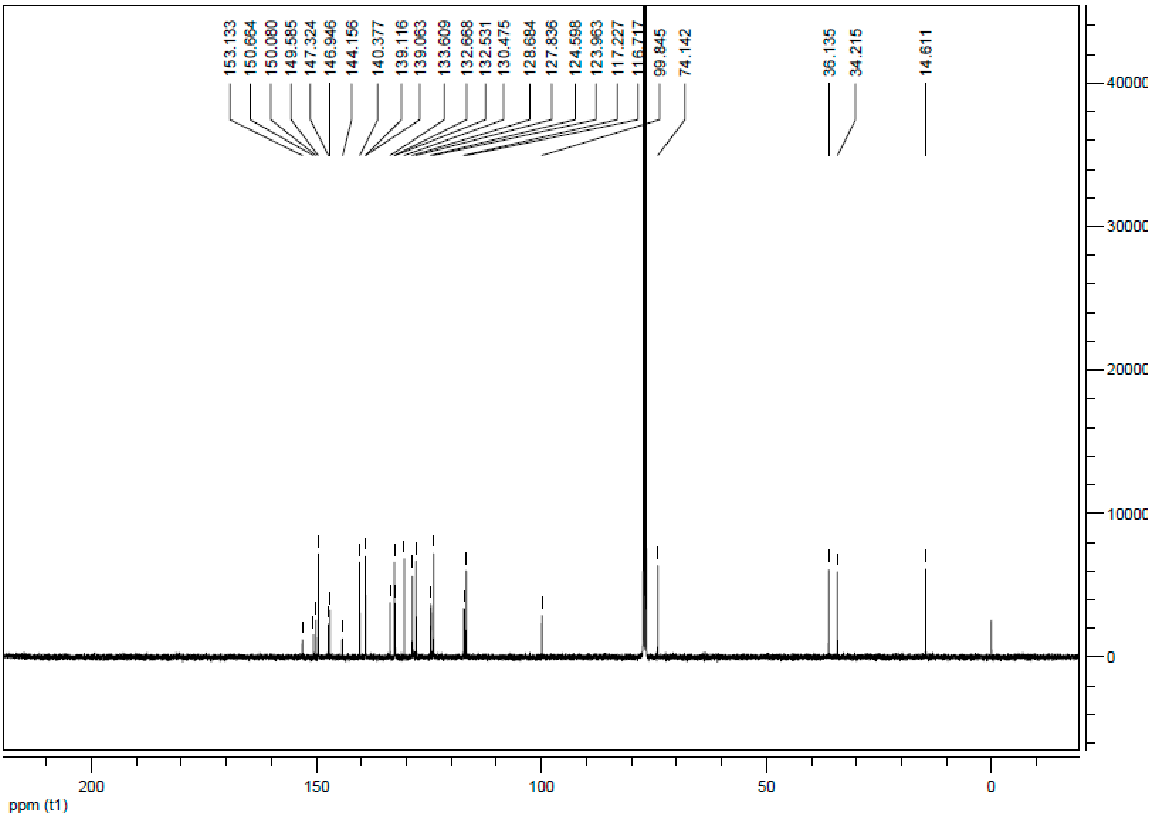

Figure S6. ^{13}C-NMR of compound **9c** (100 MHz, $CDCl_3$).

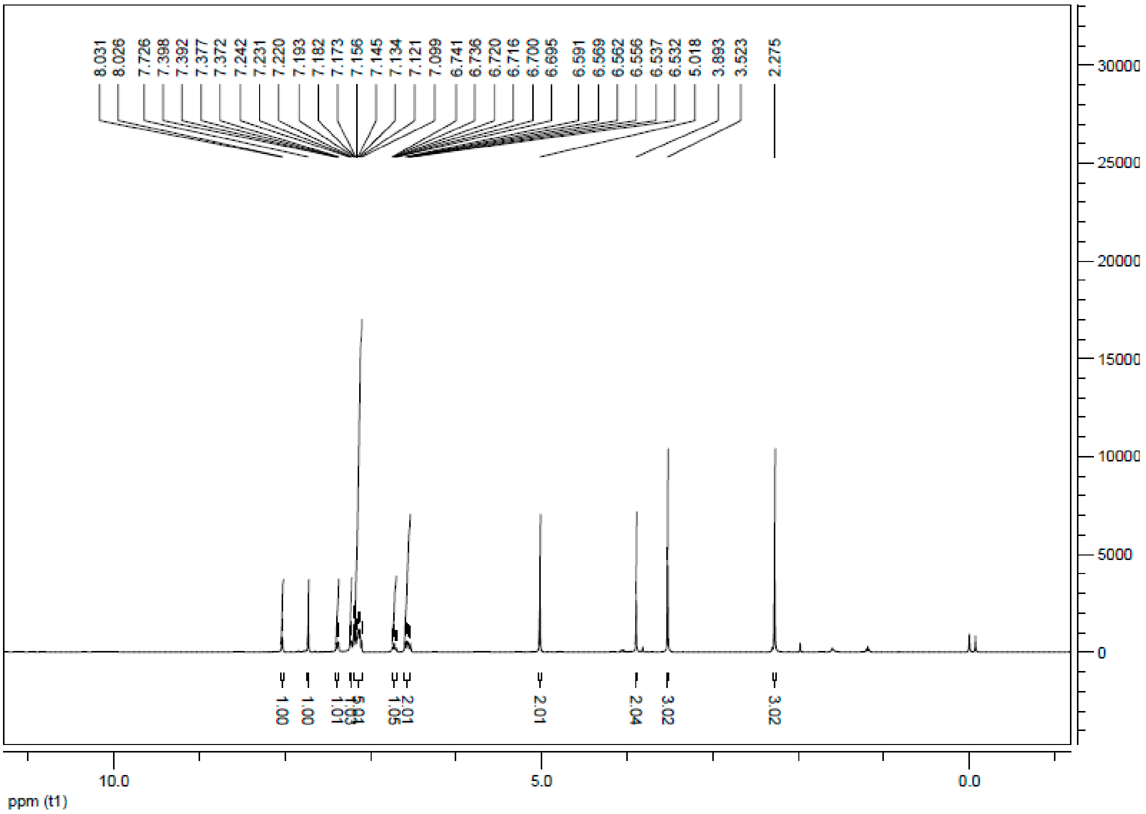

Figure S7. ^{1}H-NMR of compound **9d** (400 MHz, $CDCl_3$).

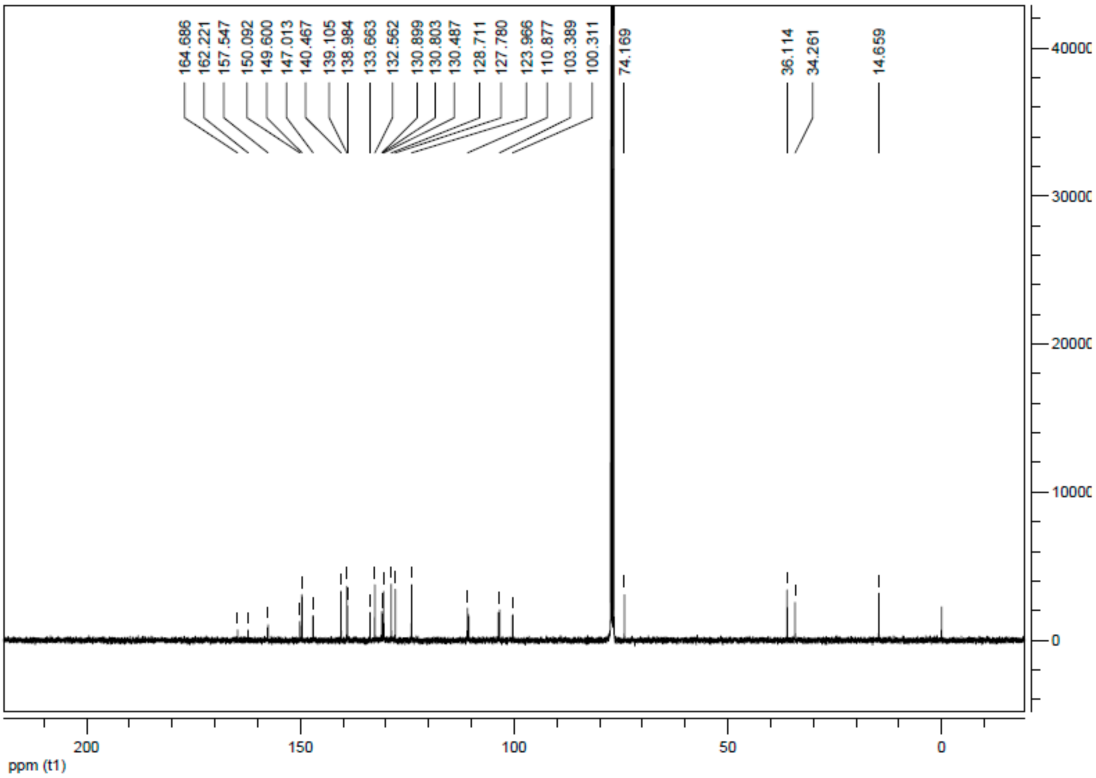

Figure S8. ^{13}C-NMR of compound **9d** (100 MHz, $CDCl_3$).

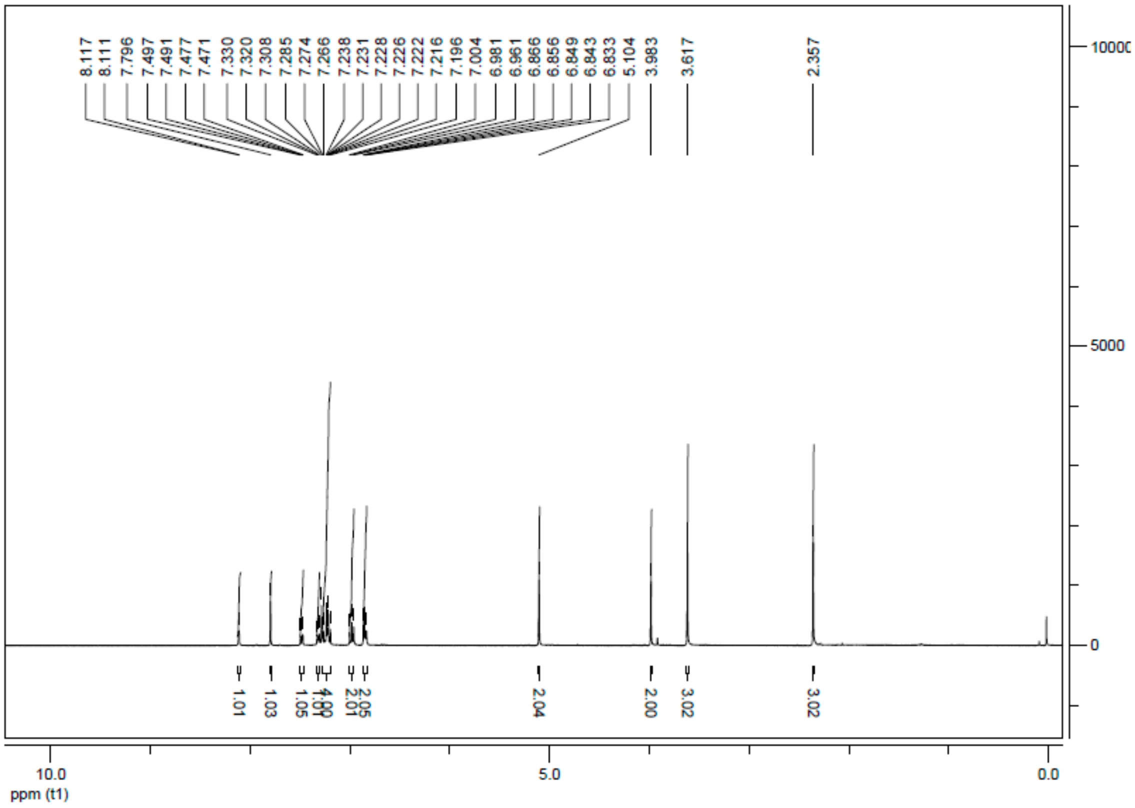

Figure S9. ^{1}H-NMR of compound **9e** (400 MHz, $CDCl_3$).

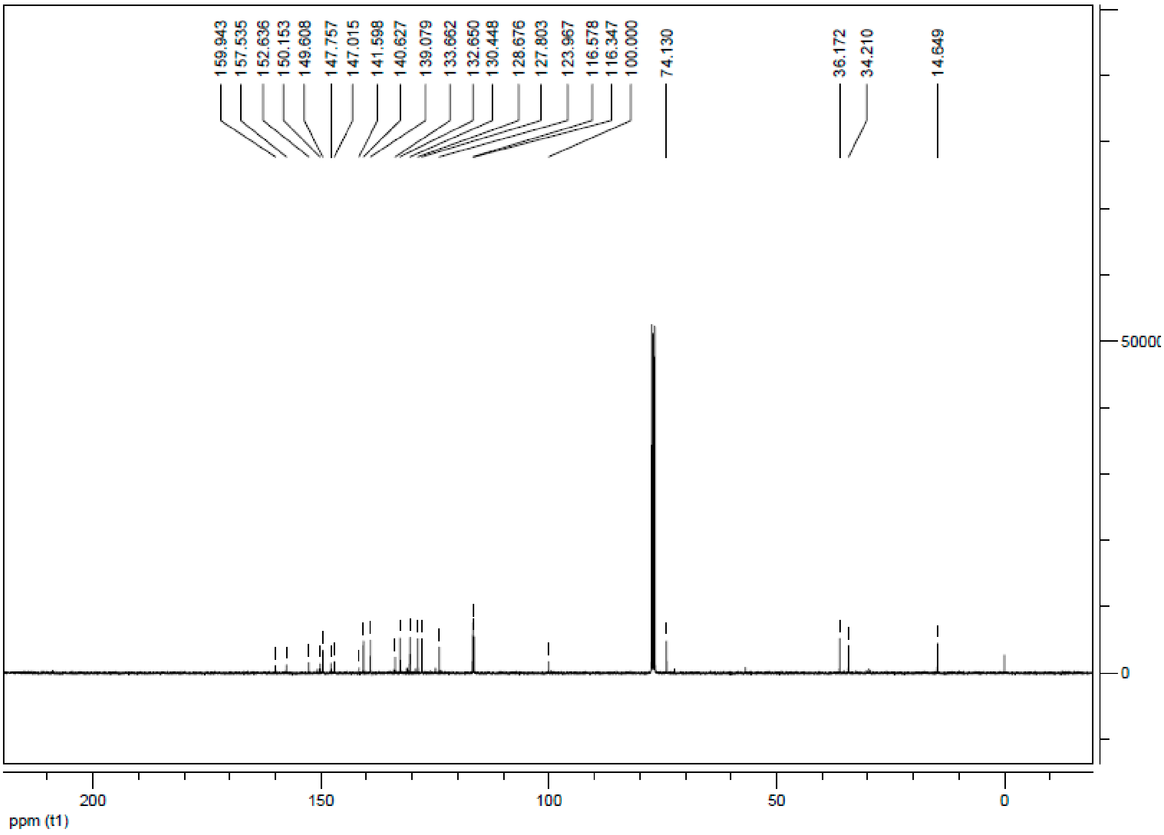

Figure S10. ^{13}C-NMR of compound **9e** (100 MHz, $CDCl_3$).

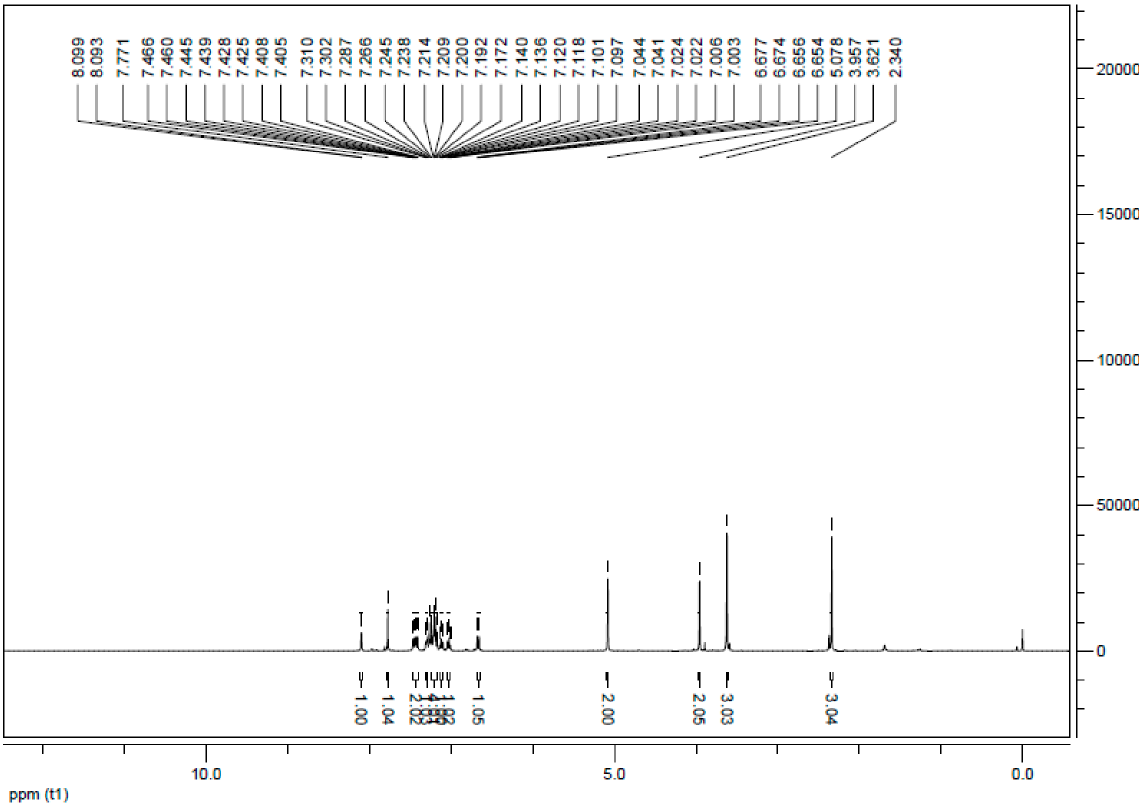

Figure S11. ^{1}H-NMR of compound **9f** (400 MHz, $CDCl_3$).

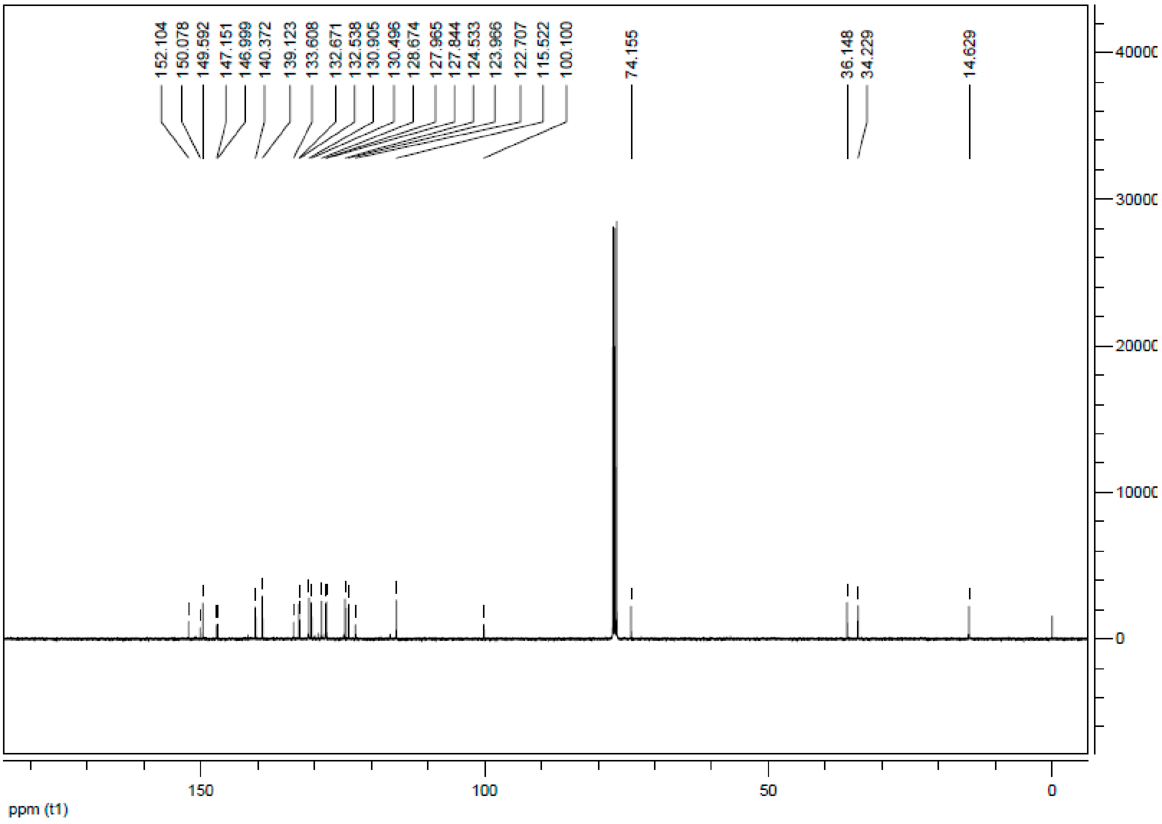

Figure S12. ^{13}C-NMR of compound **9f** (100 MHz, $CDCl_3$).

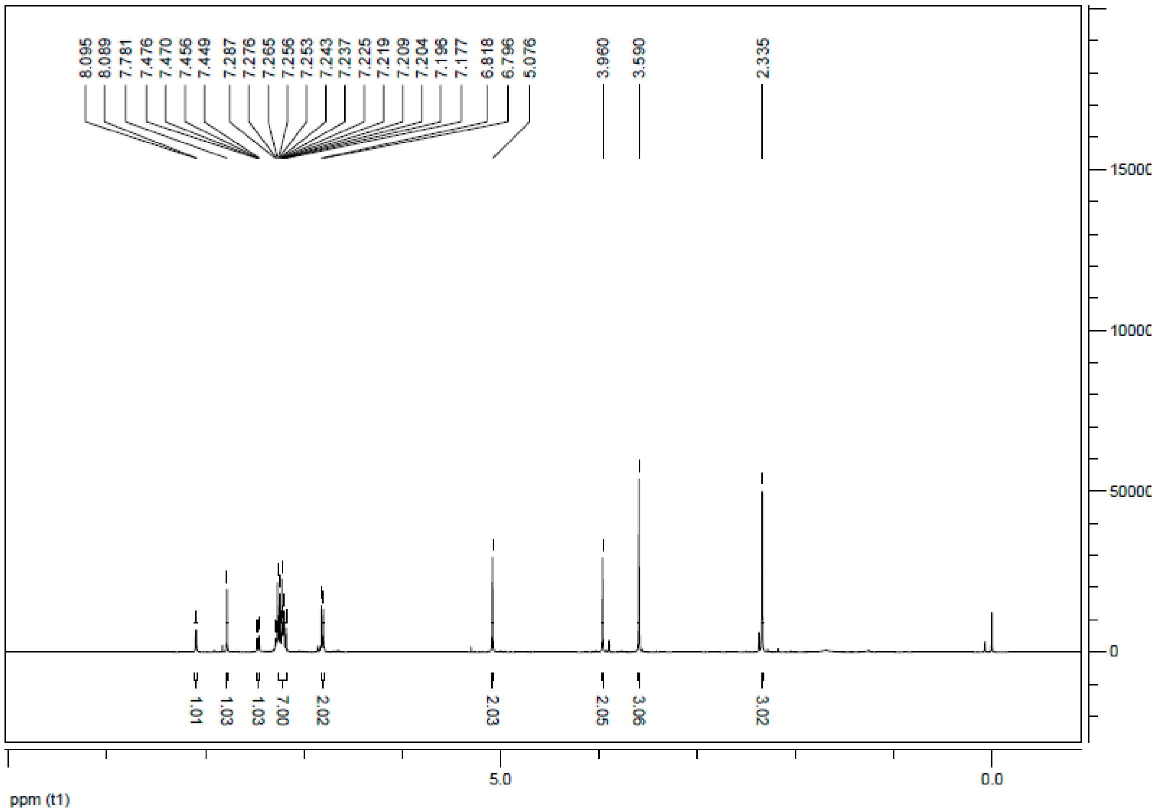

Figure S13. ^{1}H-NMR of compound **9g** (400 MHz, $CDCl_3$).

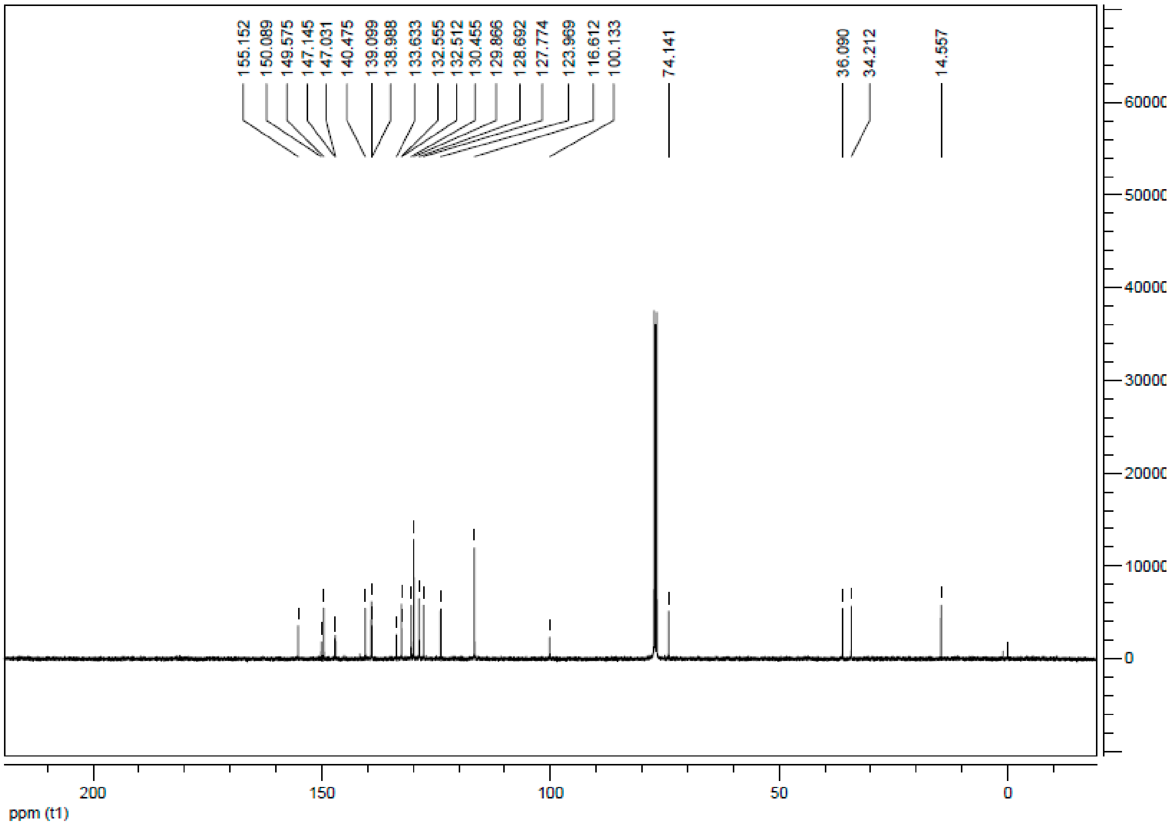

Figure S14. ^{13}C-NMR of compound **9g** (100 MHz, $CDCl_3$).

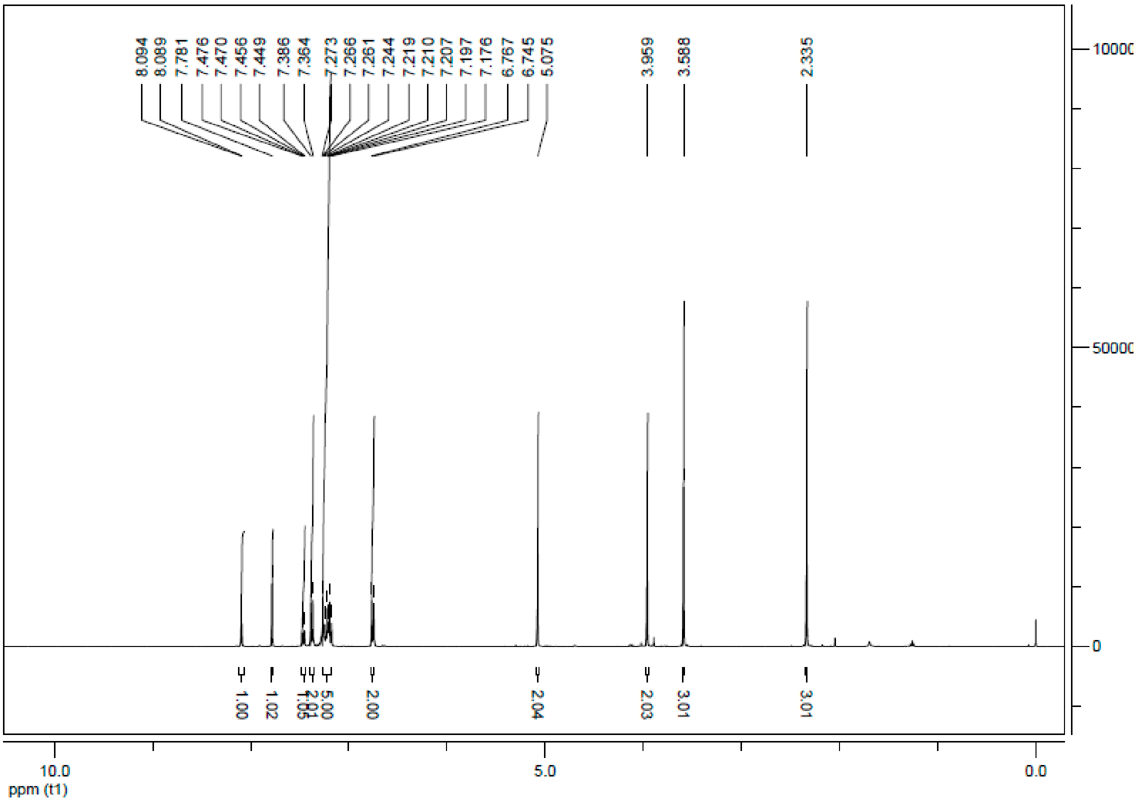

Figure S15. ^{1}H-NMR of compound **9h** (400 MHz, $CDCl_3$).

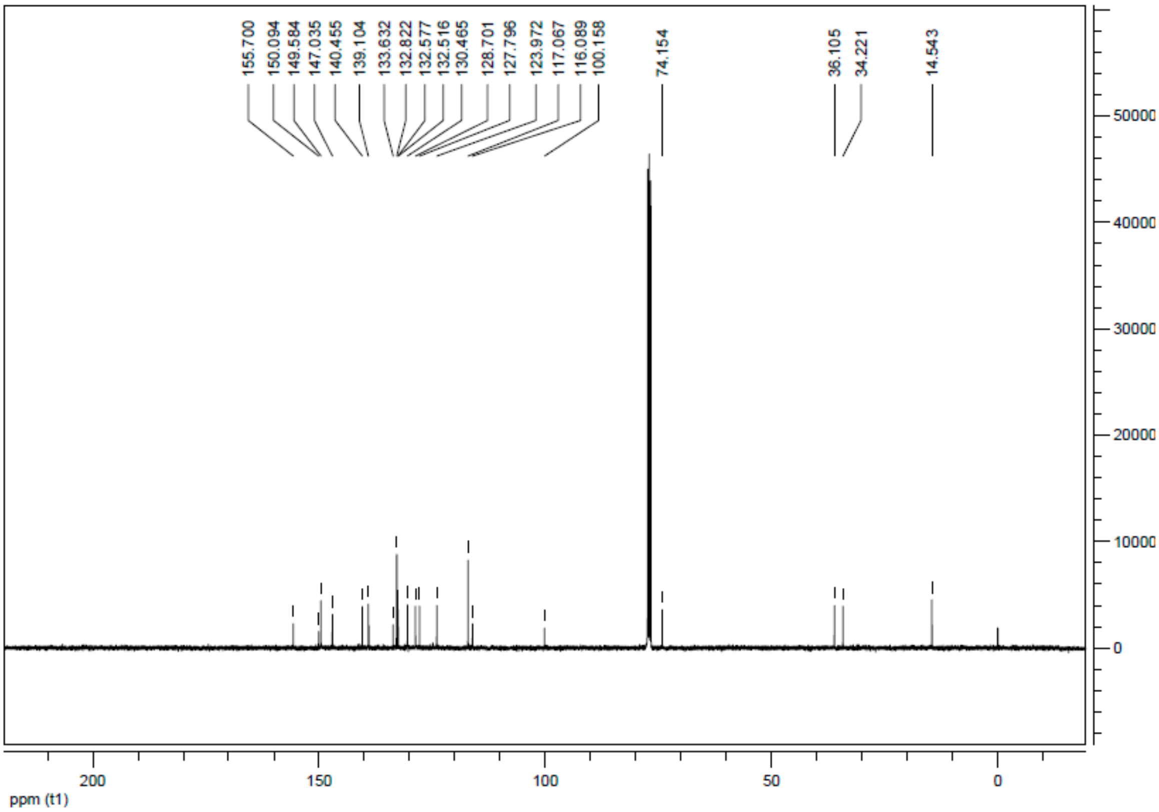

Figure S16. ^{13}C-NMR of compound **9h** (100 MHz, $CDCl_3$).

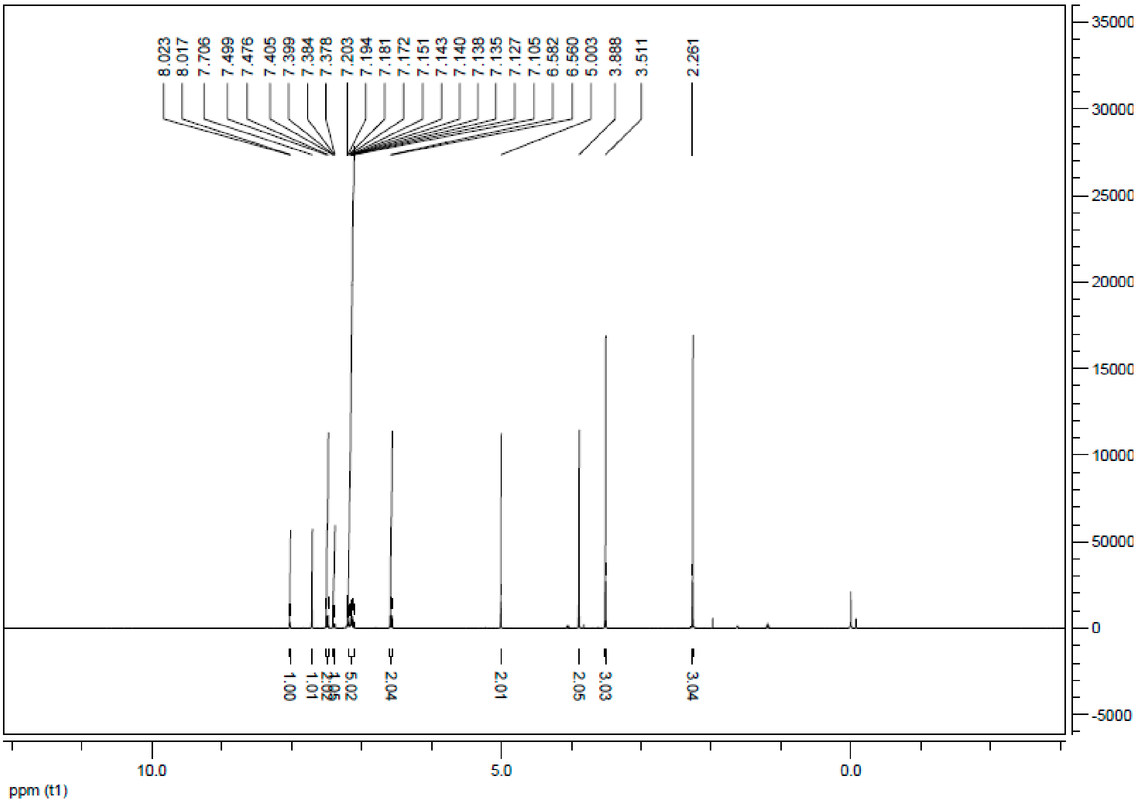

Figure S17. ^{1}H-NMR of compound **9i** (400 MHz, $CDCl_3$).

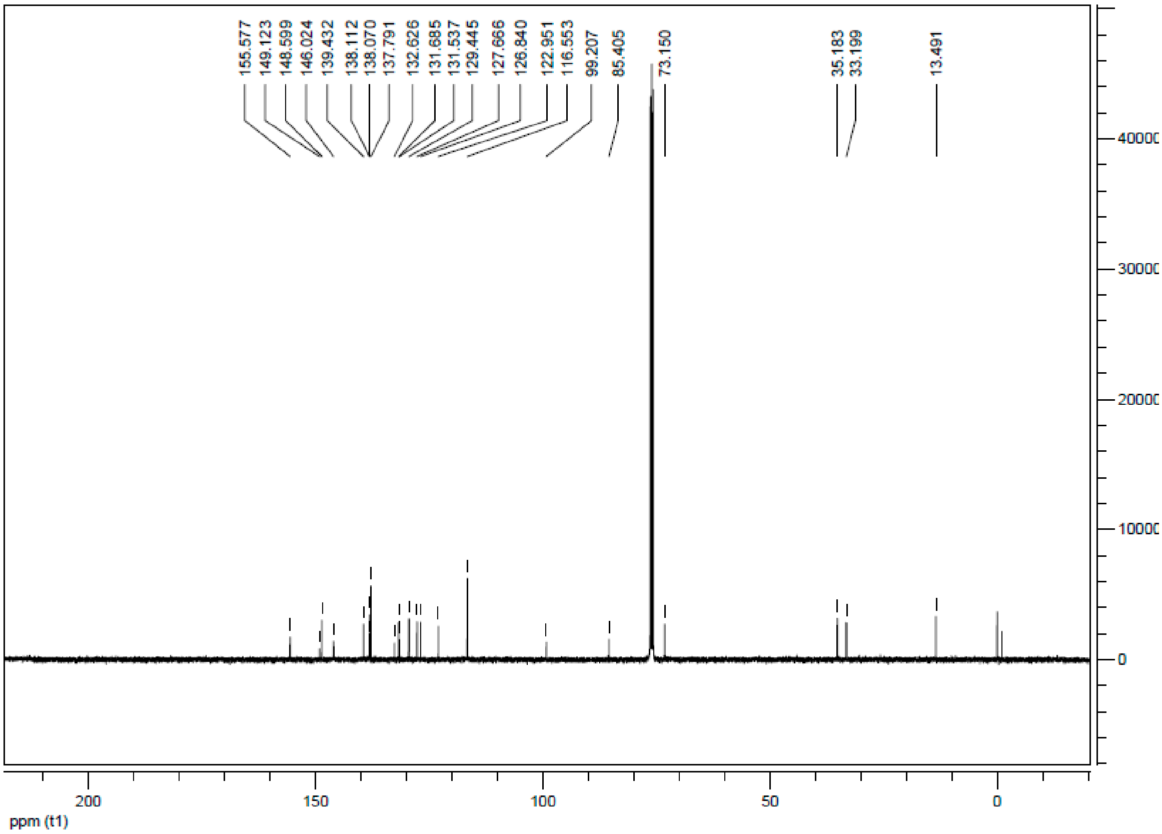

Figure S18. ^{13}C-NMR of compound **9i** (100 MHz, $CDCl_3$).

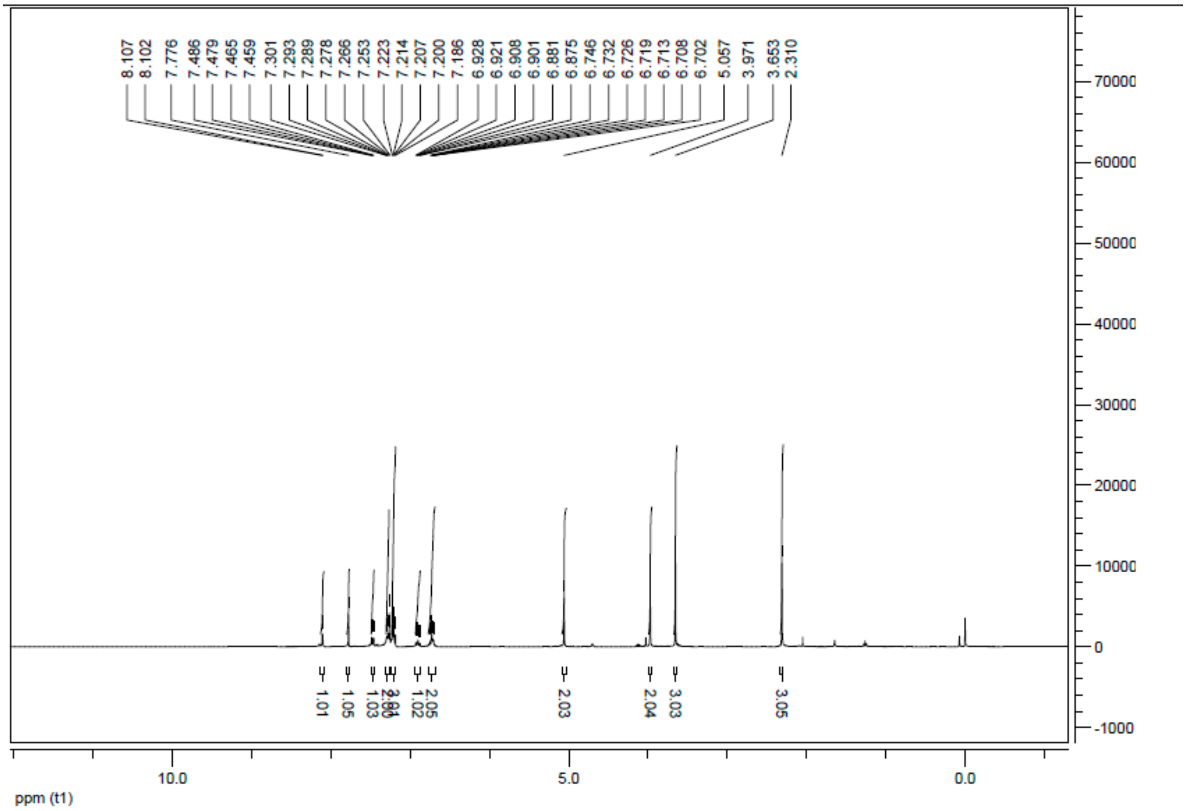

Figure S19. ^{1}H-NMR of compound **9j** (400 MHz, $CDCl_3$).

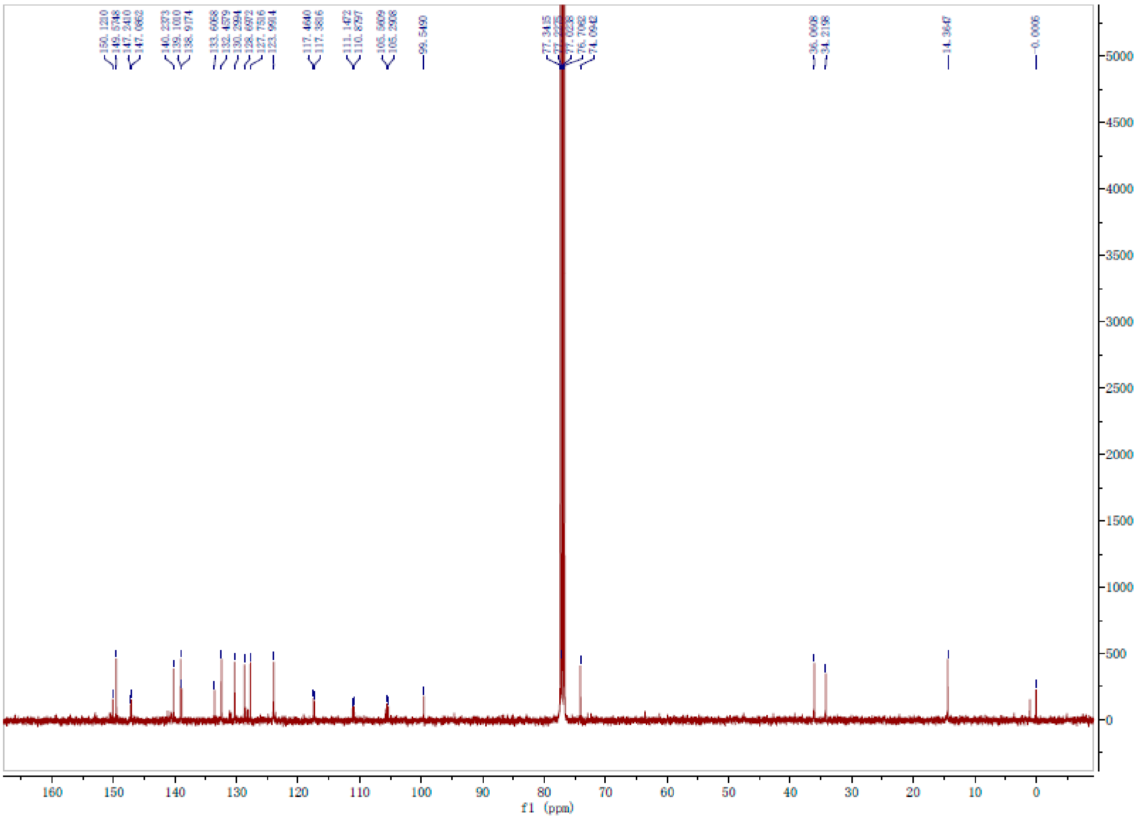

Figure S20. ^{13}C-NMR of compound **9j** (100 MHz, $CDCl_3$).

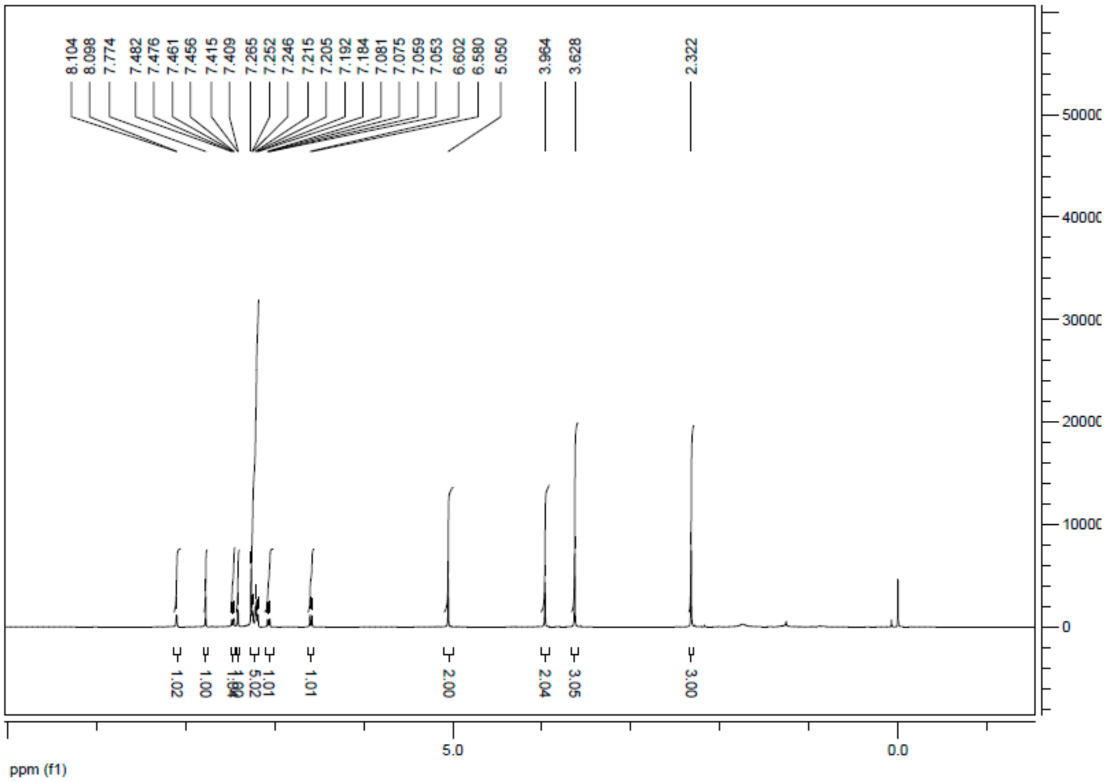

Figure S21. ^{1}H-NMR of compound **9k** (400 MHz, $CDCl_3$).

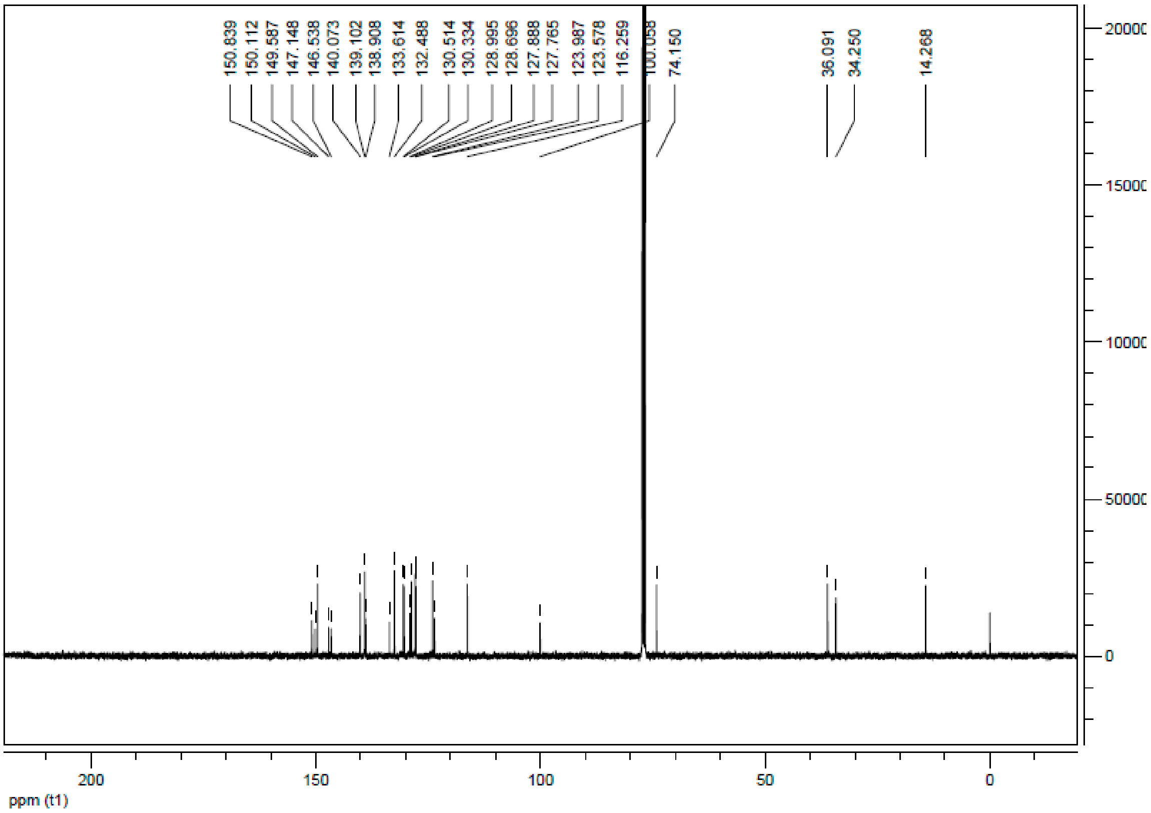

Figure S22. ^{13}C-NMR of compound **9k** (100 MHz, $CDCl_3$).

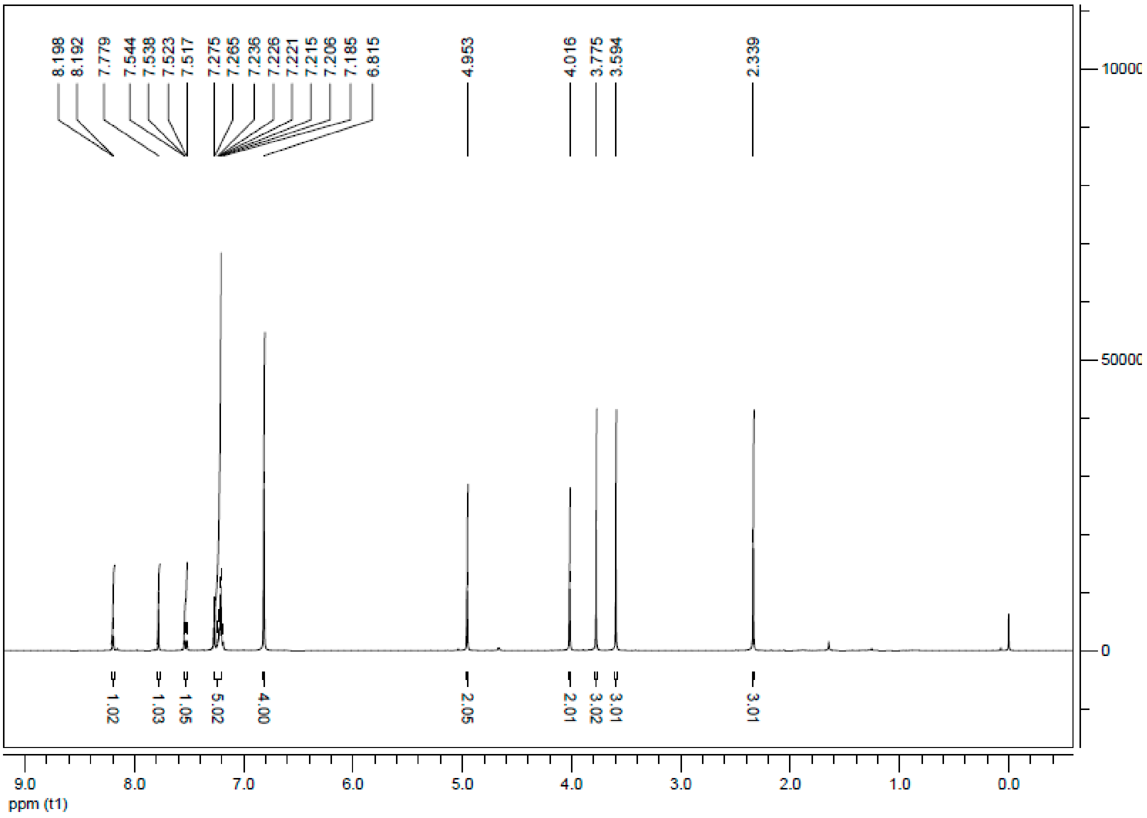

Figure S23. ^{1}H-NMR of compound **9l** (400 MHz, $CDCl_3$).

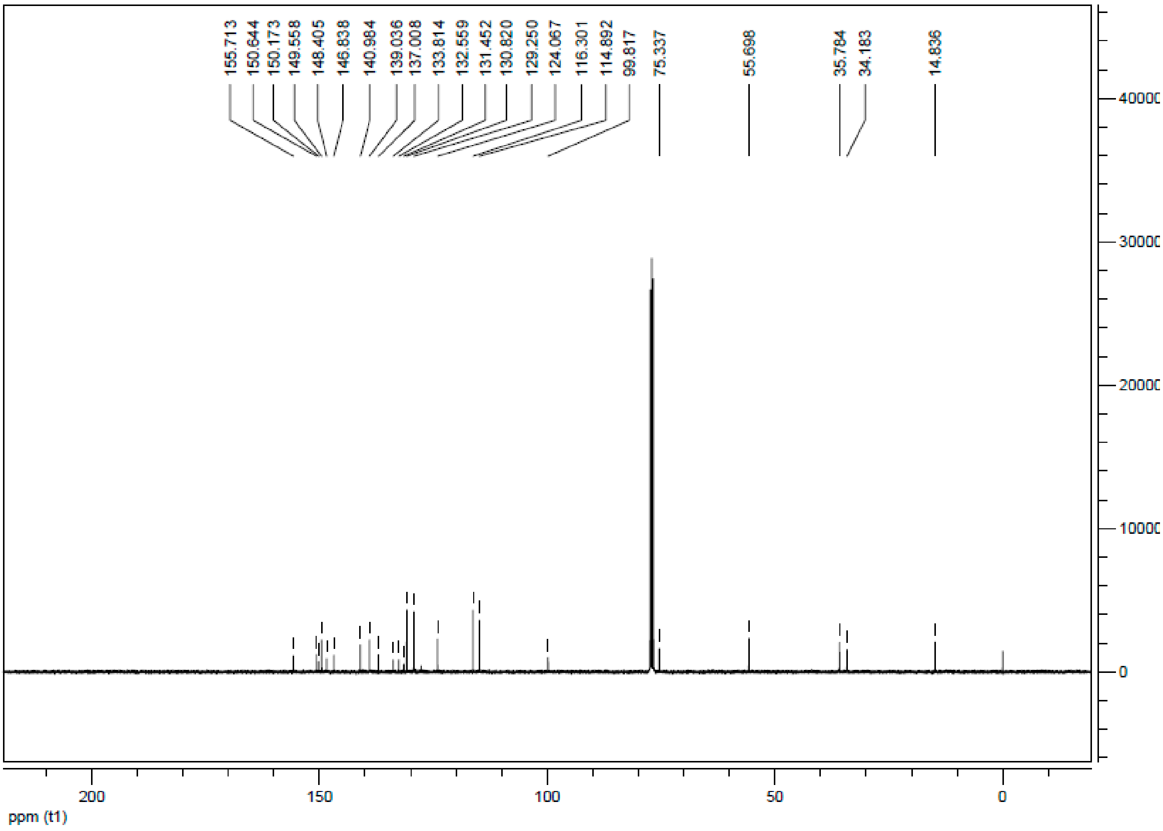

Figure S24. ^{13}C-NMR of compound **9l** (100 MHz, $CDCl_3$).

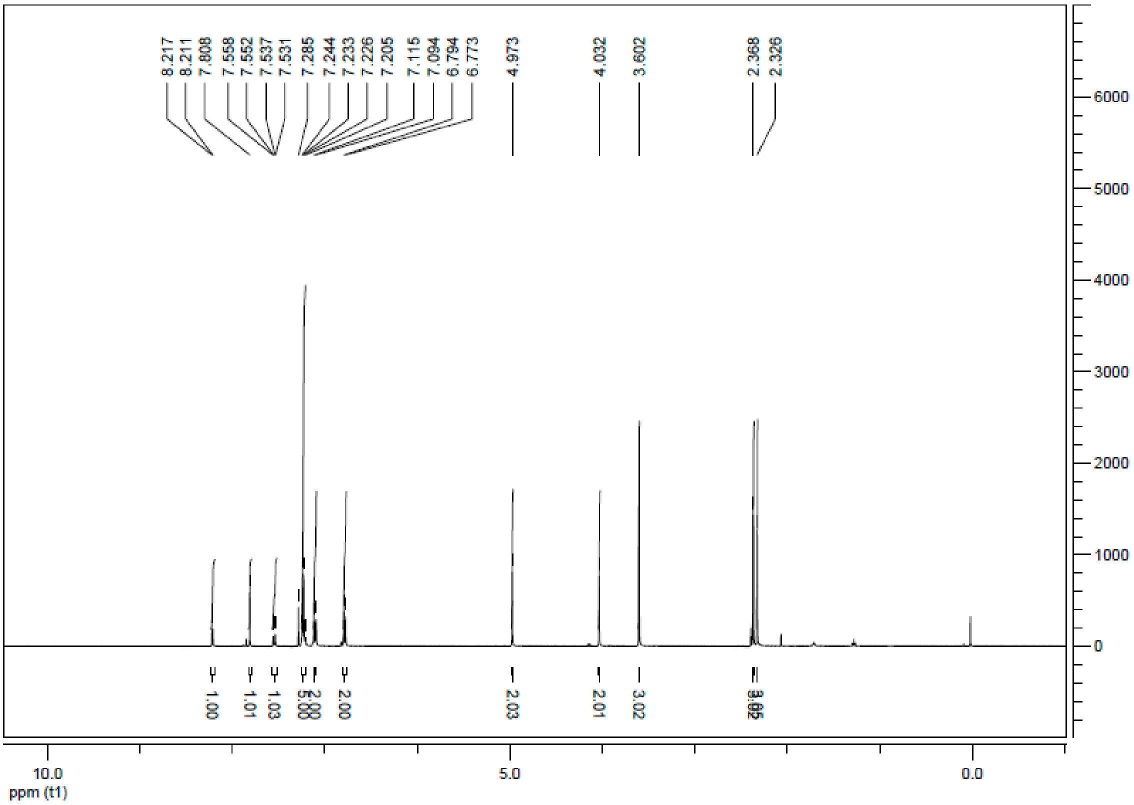

Figure S25. ^{1}H-NMR of compound **9m** (400 MHz, $CDCl_3$).

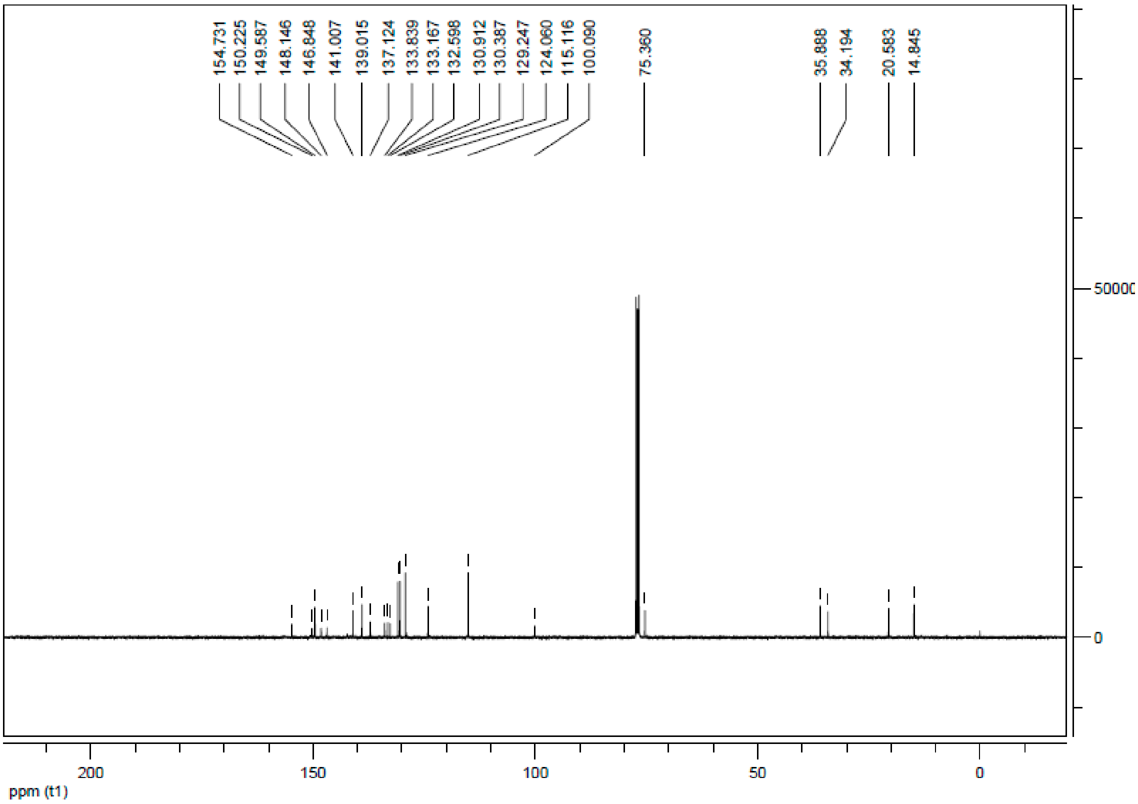

Figure S26. ^{13}C-NMR of compound **9m** (100 MHz, $CDCl_3$).

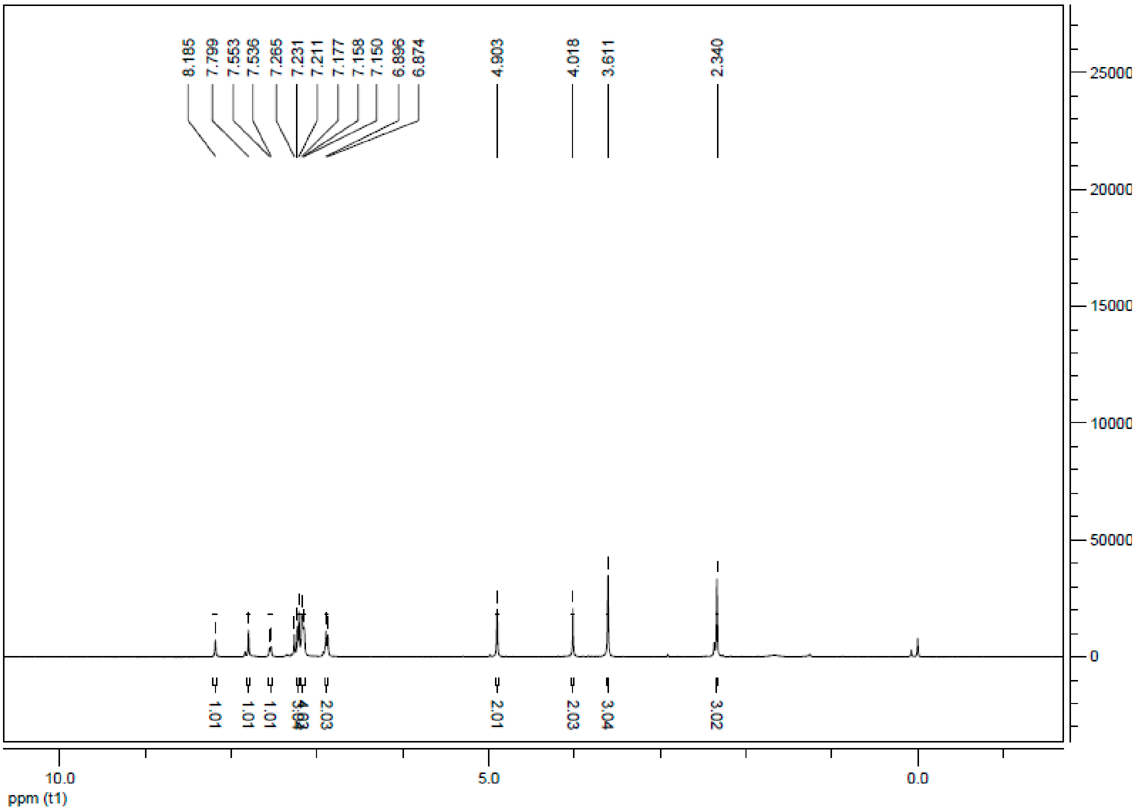

Figure S27. ^{1}H-NMR of compound **9n** (400 MHz, $CDCl_3$).

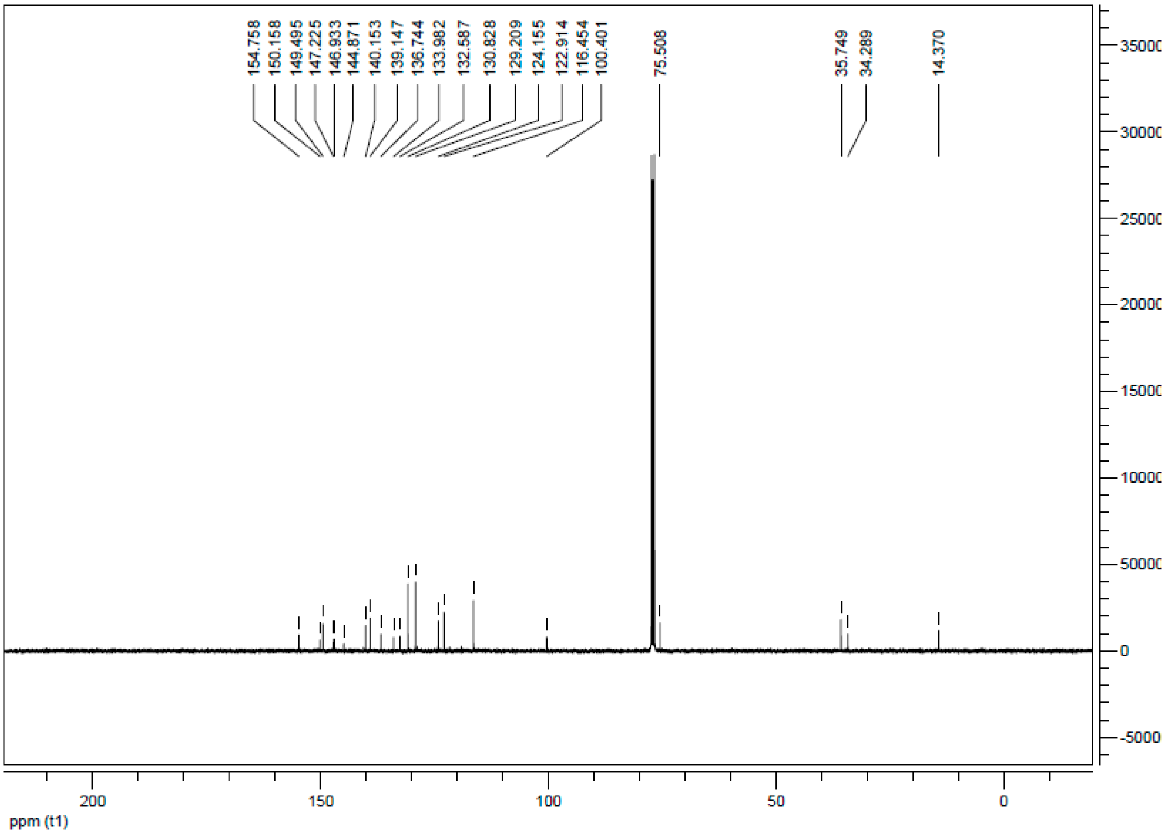

Figure S28. ^{13}C-NMR of compound **9n** (100 MHz, $CDCl_3$).

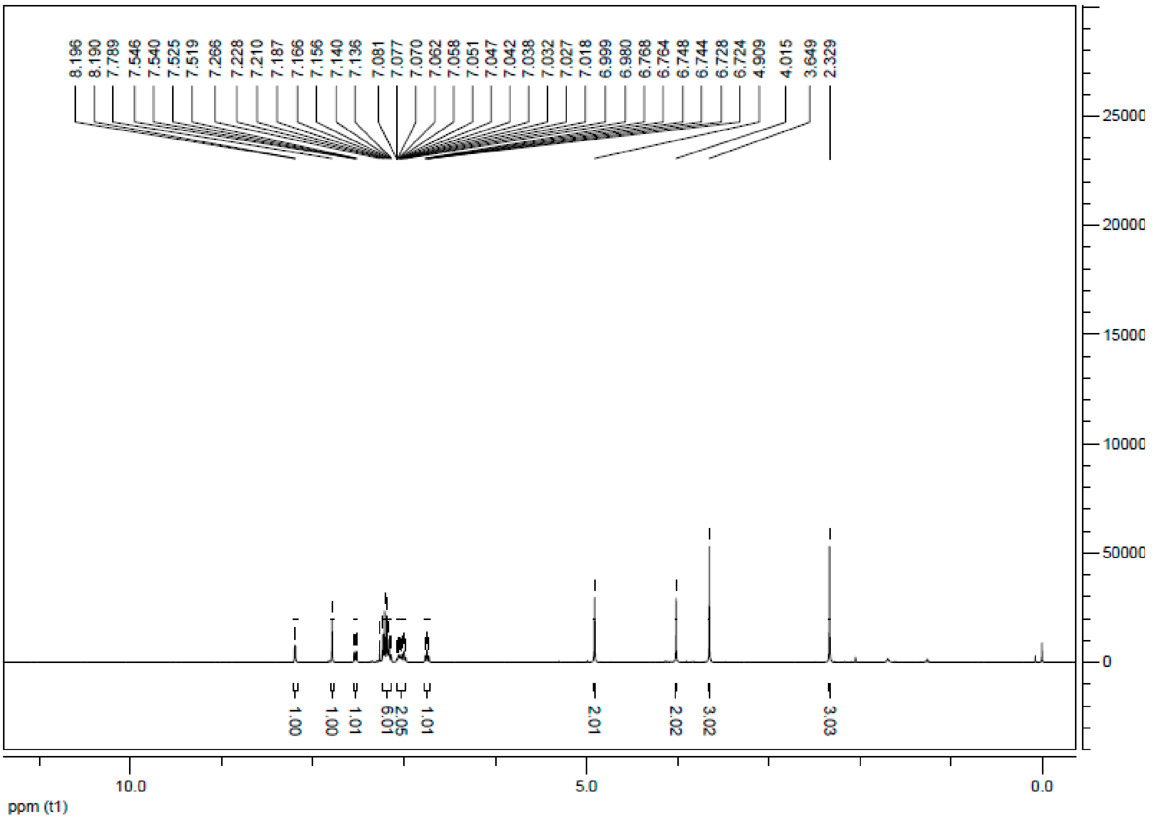

Figure S29. ^{1}H-NMR of compound **9o** (400 MHz, $CDCl_3$).

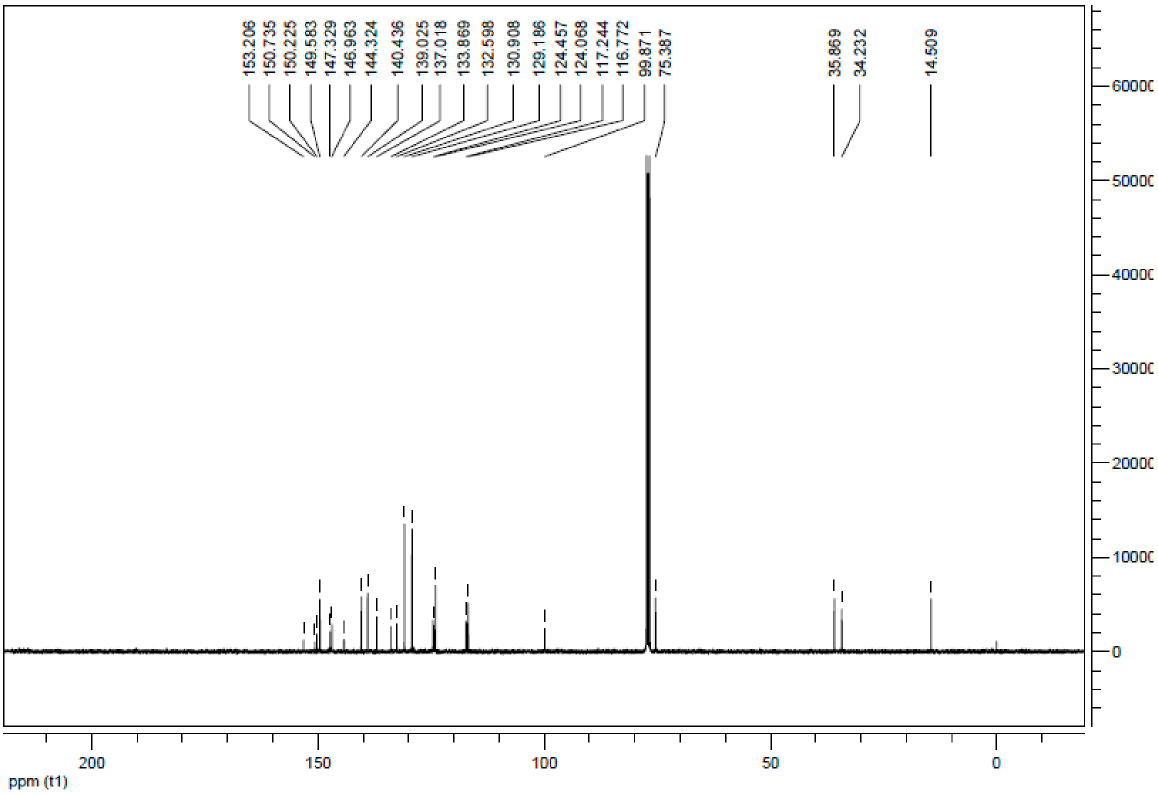

Figure S30. ^{13}C-NMR of compound **9o** (100 MHz, $CDCl_3$).

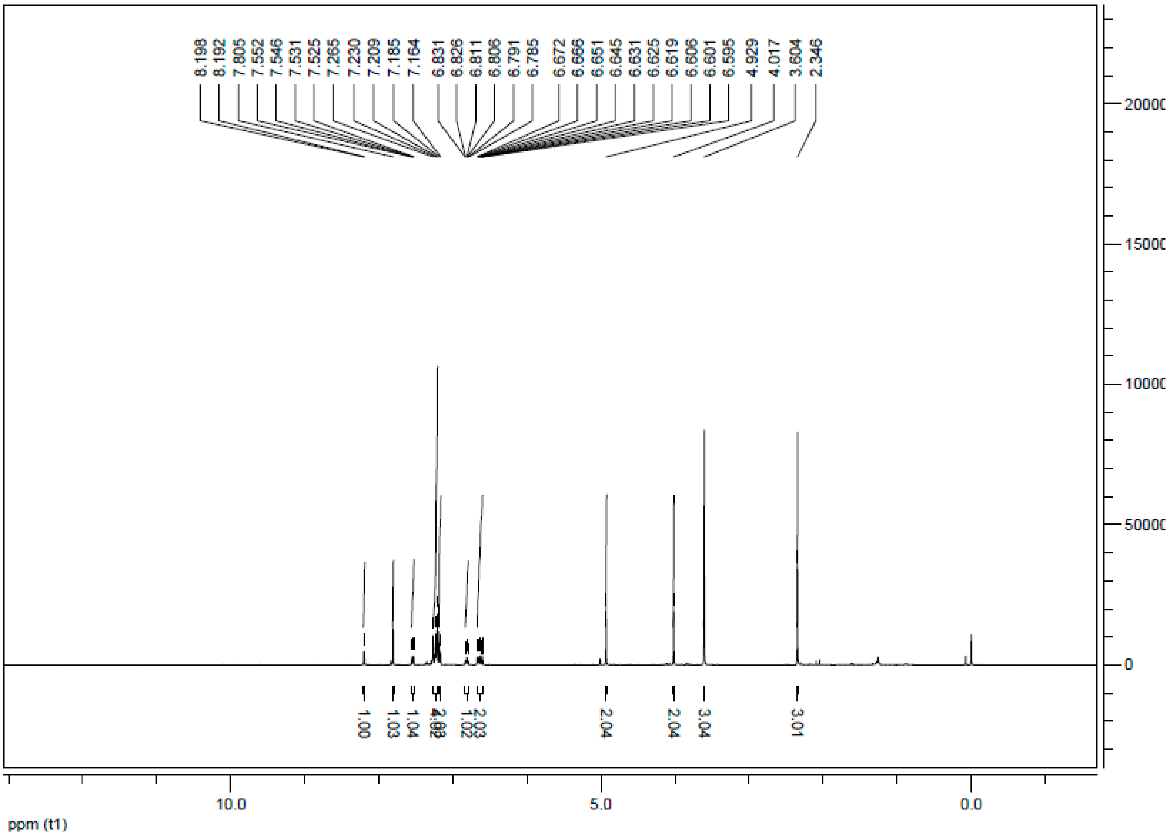

Figure S31. ^{1}H-NMR of compound **9p** (400 MHz, $CDCl_3$).

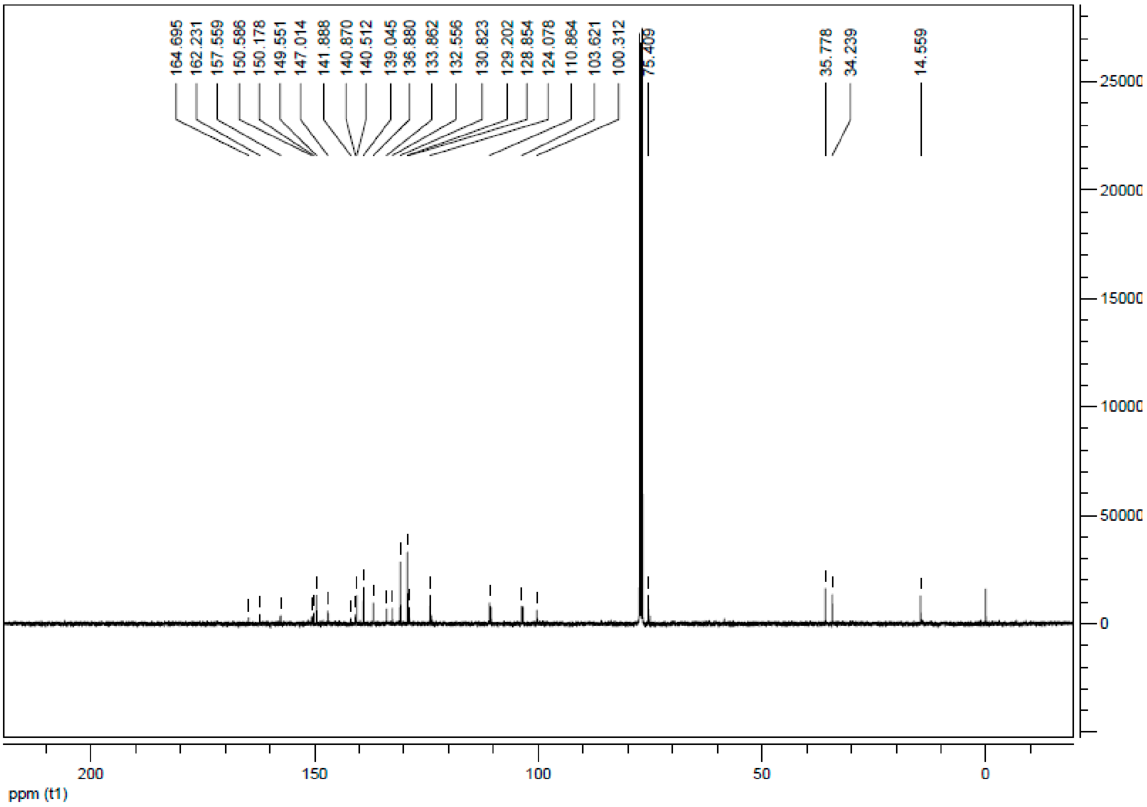

Figure S32. ^{13}C-NMR of compound **9p** (100 MHz, $CDCl_3$).

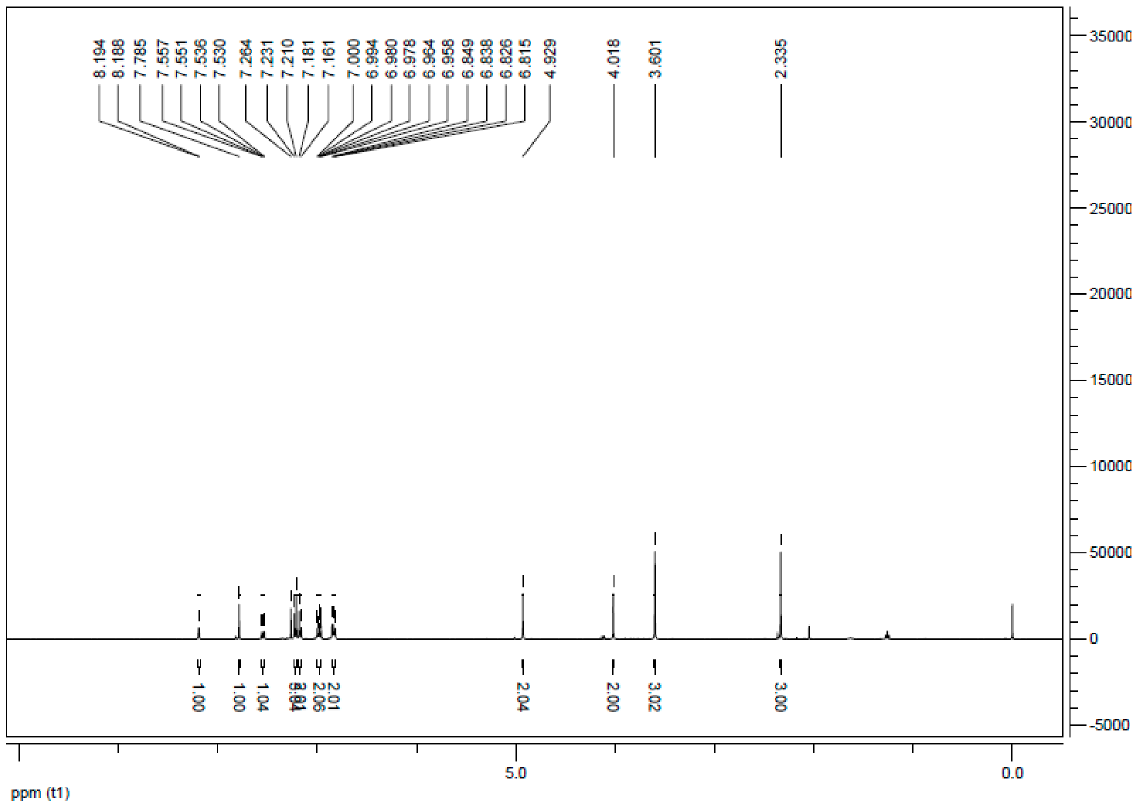

Figure S33. ^{1}H-NMR of compound **9q** (400 MHz, $CDCl_3$).

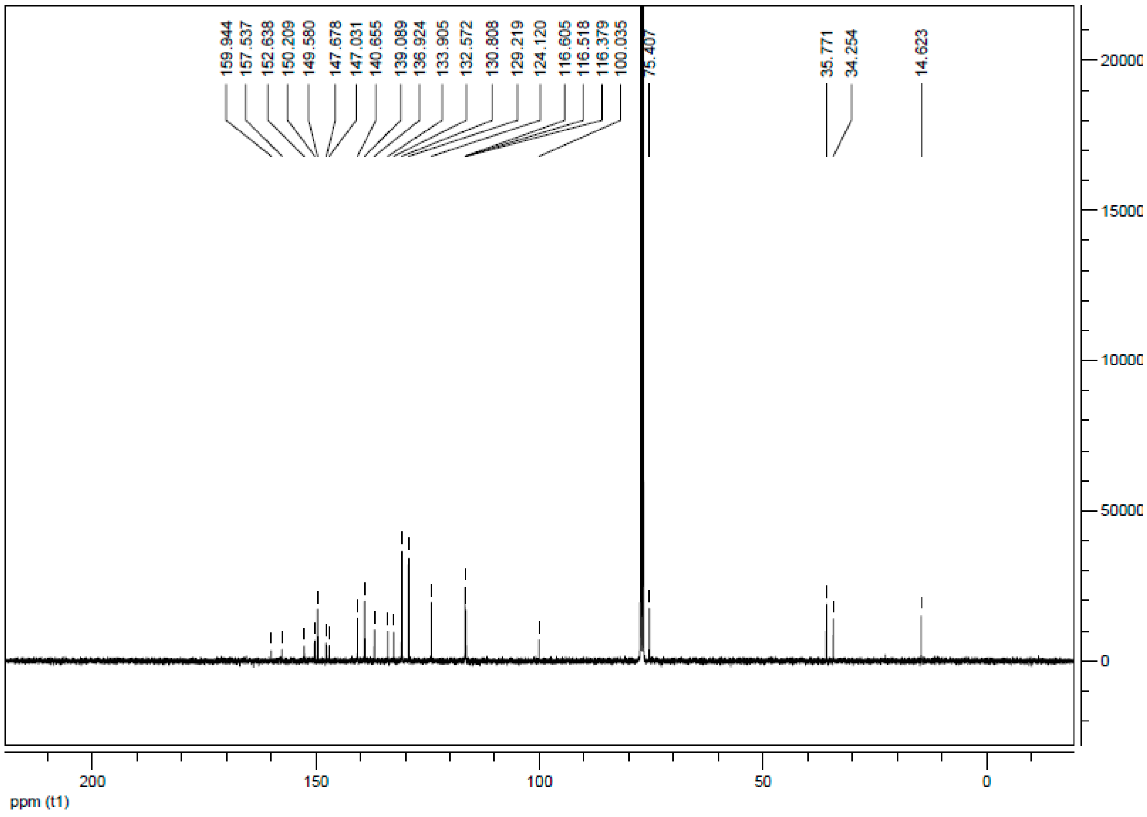

Figure S34. ^{13}C-NMR of compound **9q** (100 MHz, $CDCl_3$).

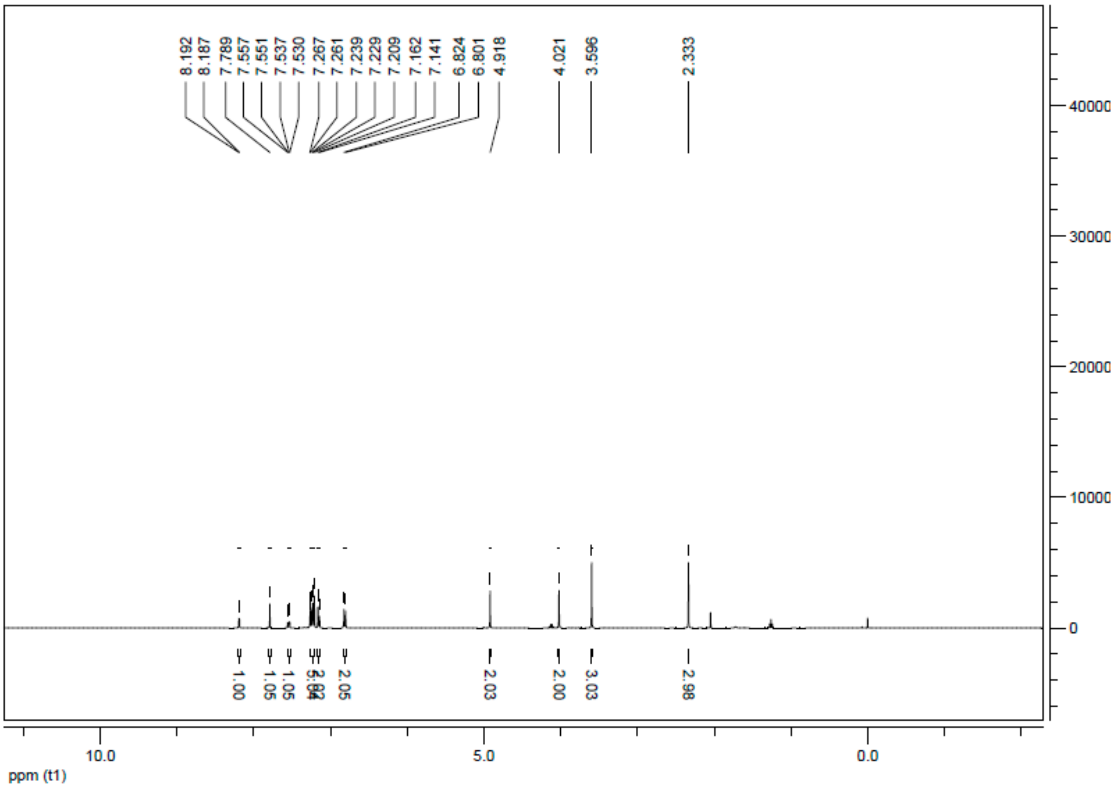

Figure S35. ^{1}H-NMR of compound **9r** (400 MHz, $CDCl_3$).

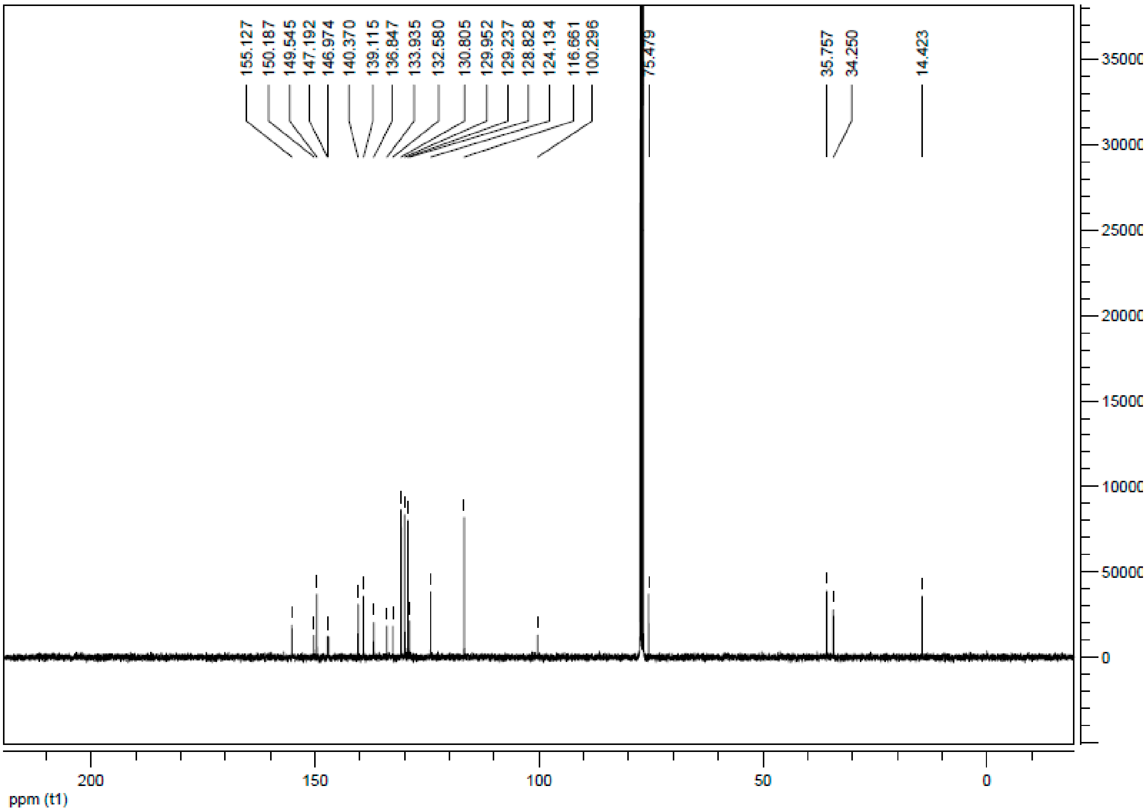

Figure S36. ^{13}C-NMR of compound **9r** (100 MHz, $CDCl_3$).

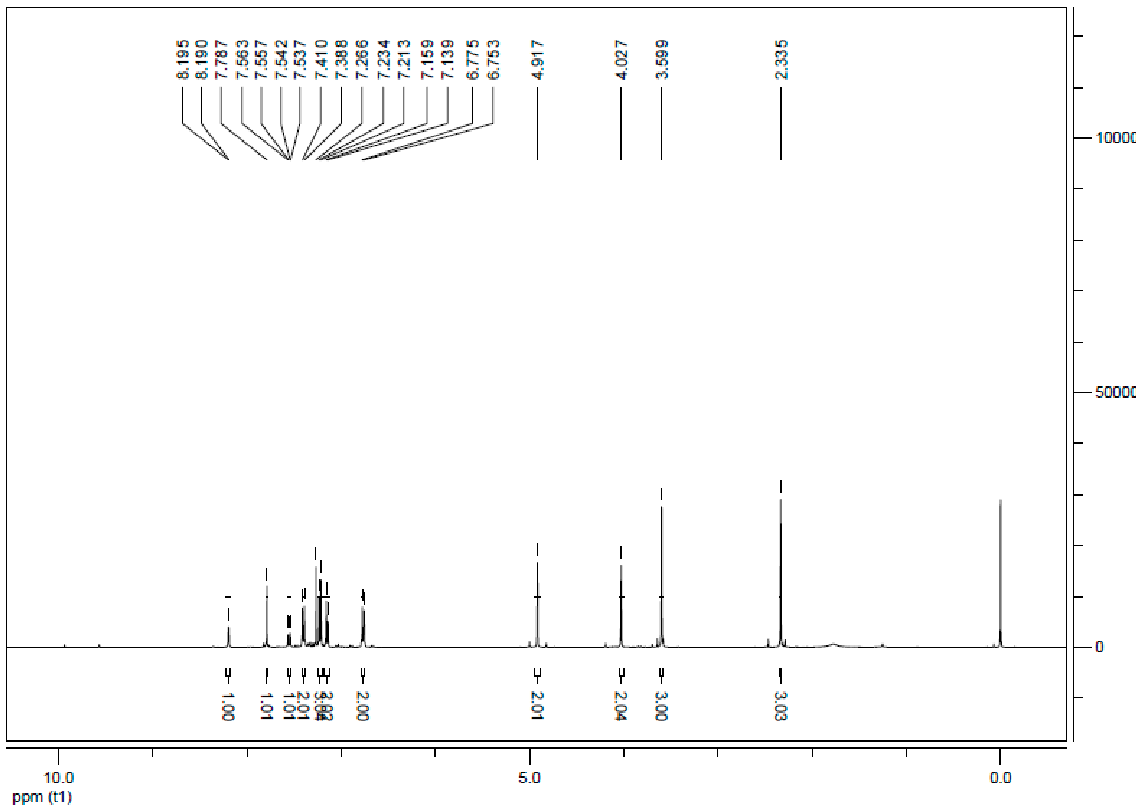

Figure S37. ^{1}H-NMR of compound **9s** (400 MHz, $CDCl_3$).

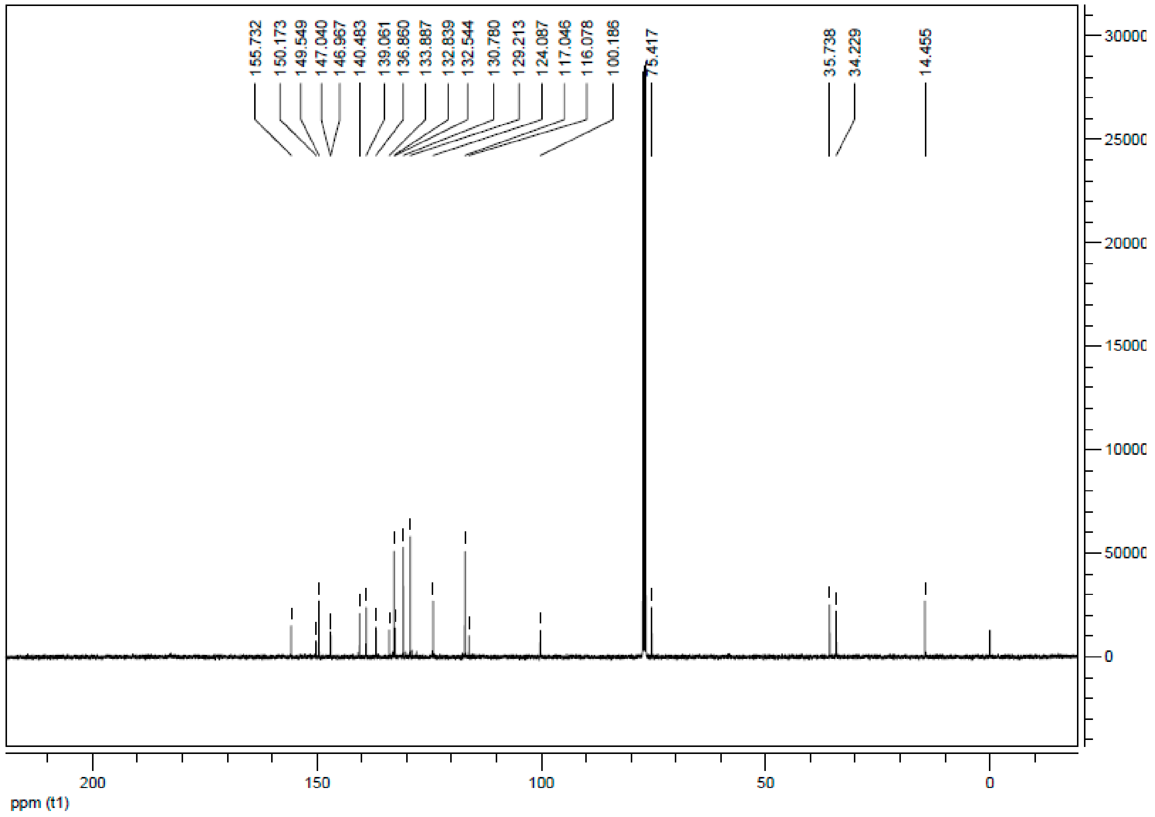

Figure S38. ^{13}C-NMR of compound **9s** (100 MHz, $CDCl_3$).

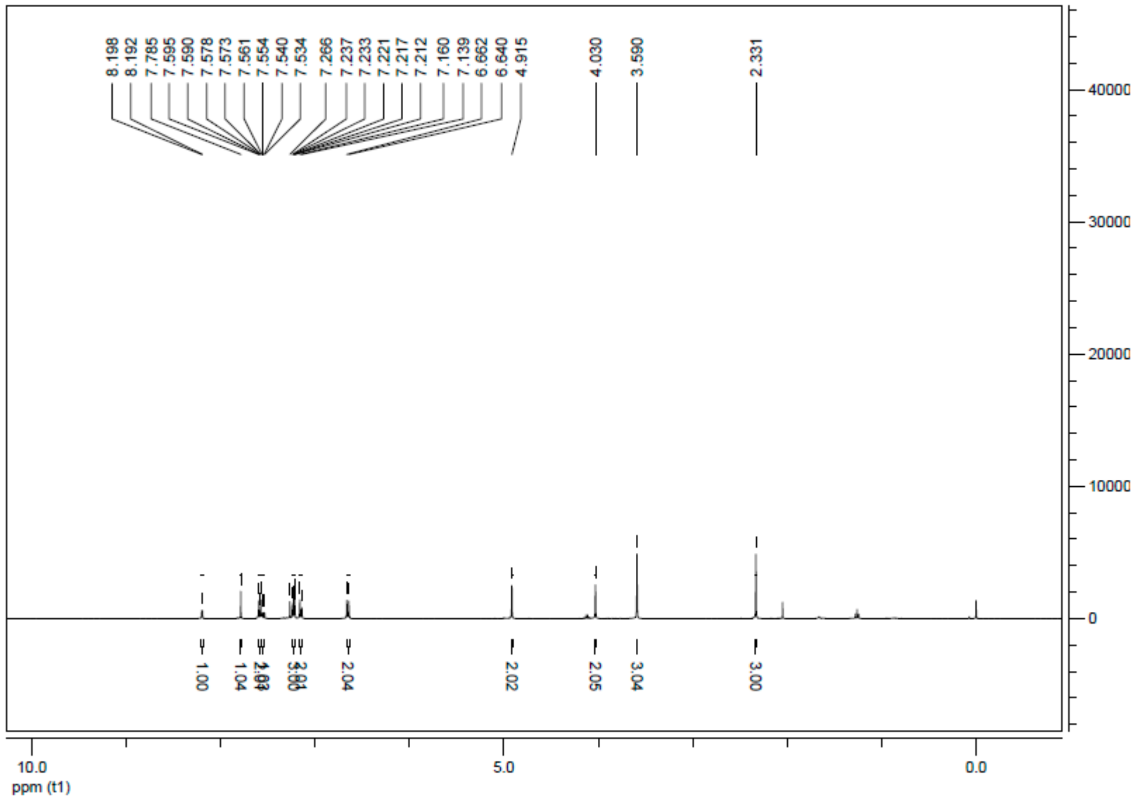

Figure S39. ^{1}H-NMR of compound **9t** (400 MHz, $CDCl_3$).

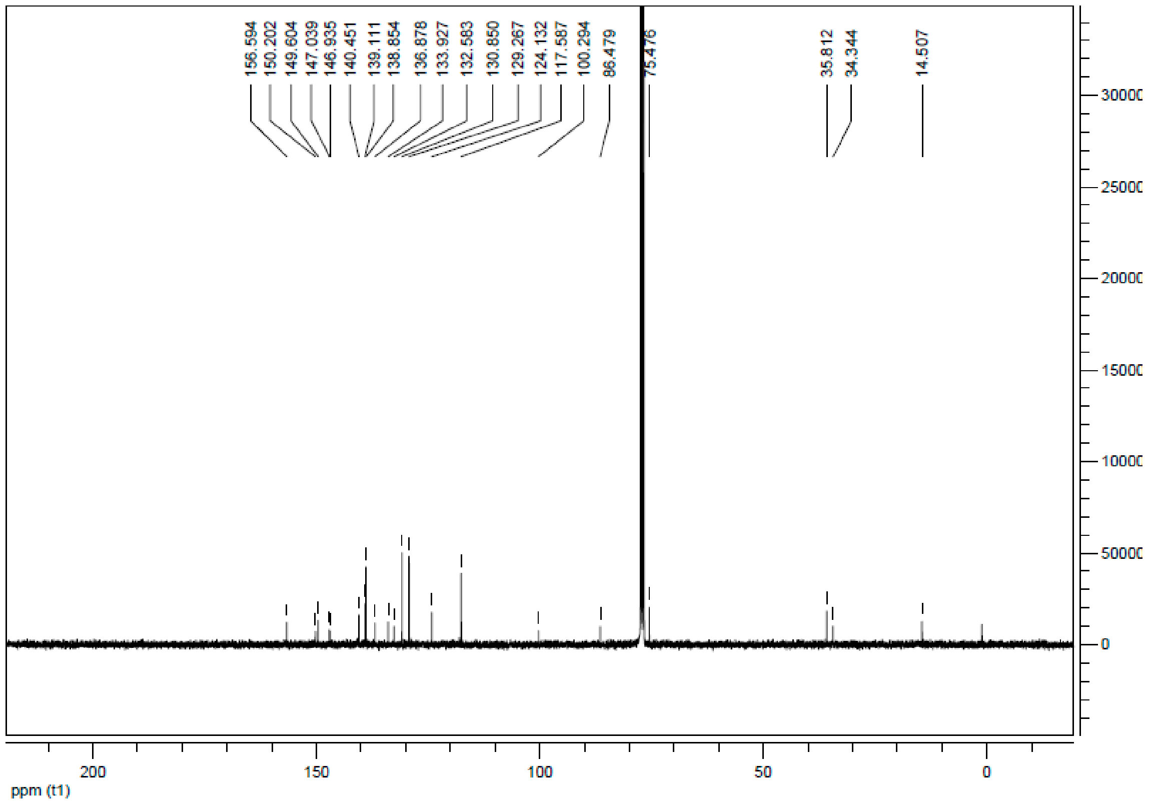

Figure S40. ^{13}C-NMR of compound **9t** (100 MHz, $CDCl_3$).

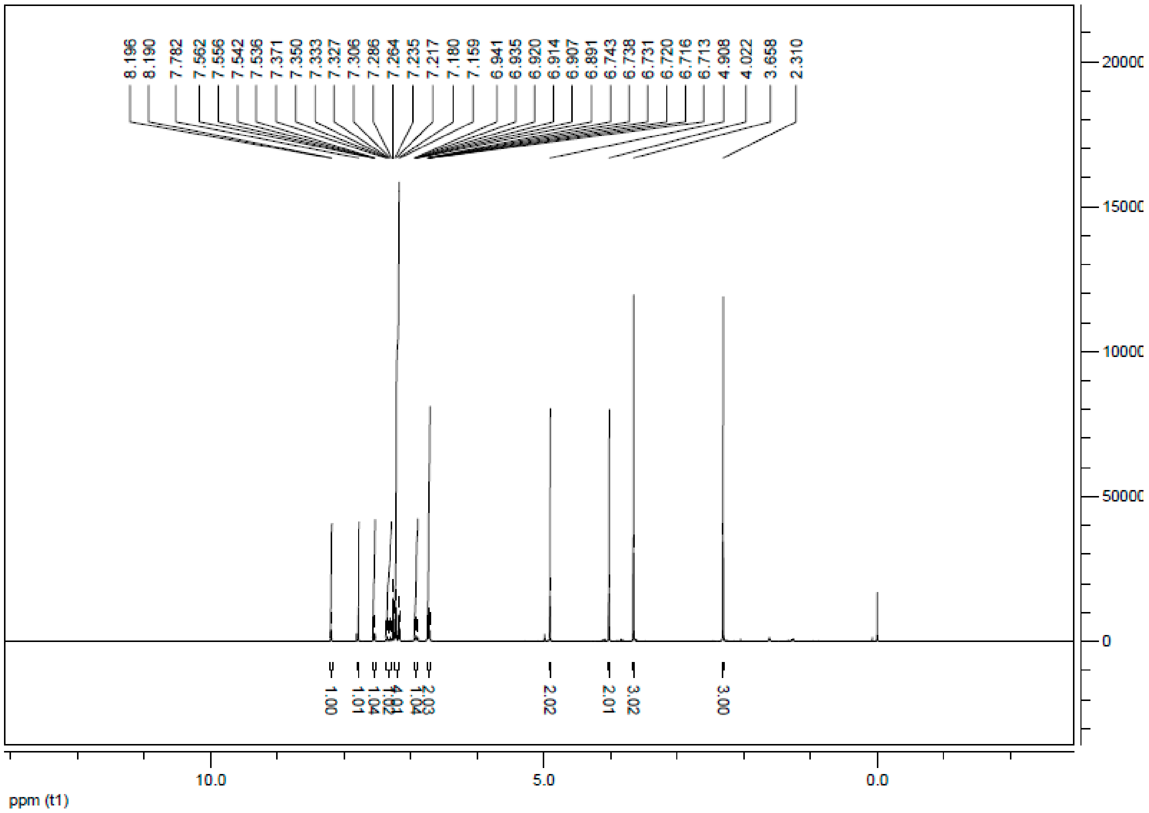

Figure S41. ^{1}H-NMR of compound **9u** (400 MHz, $CDCl_3$).

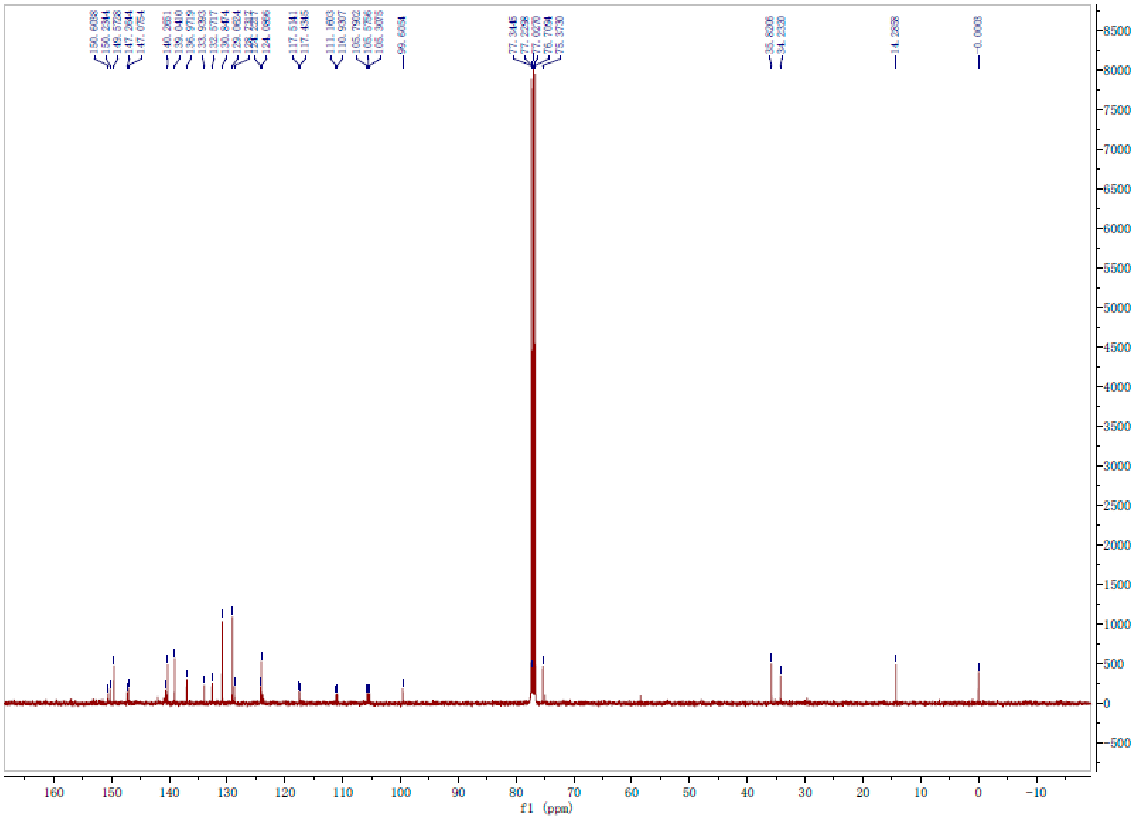

Figure S42. ^{13}C-NMR of compound **9u** (100 MHz, $CDCl_3$).

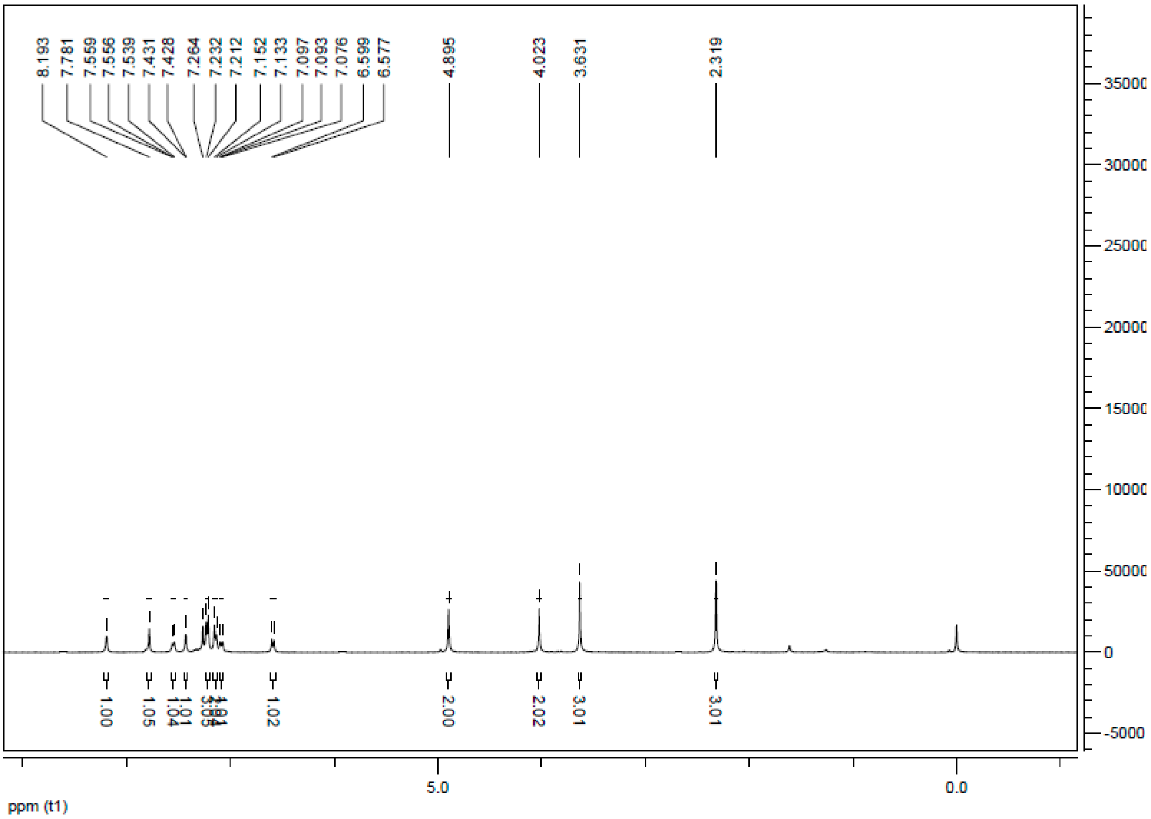

Figure S43. ^{1}H-NMR of compound **9v** (400 MHz, $CDCl_3$).

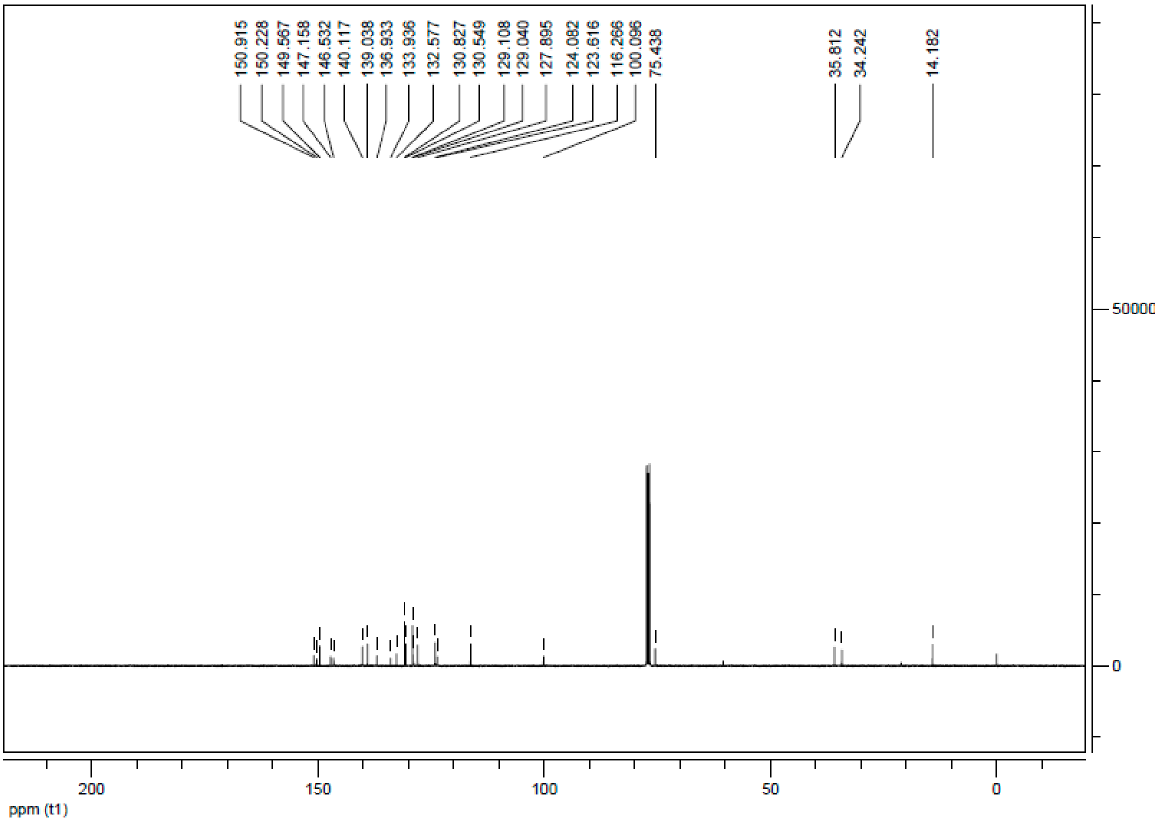

Figure S44. ^{13}C-NMR of compound **9v** (100 MHz, $CDCl_3$).

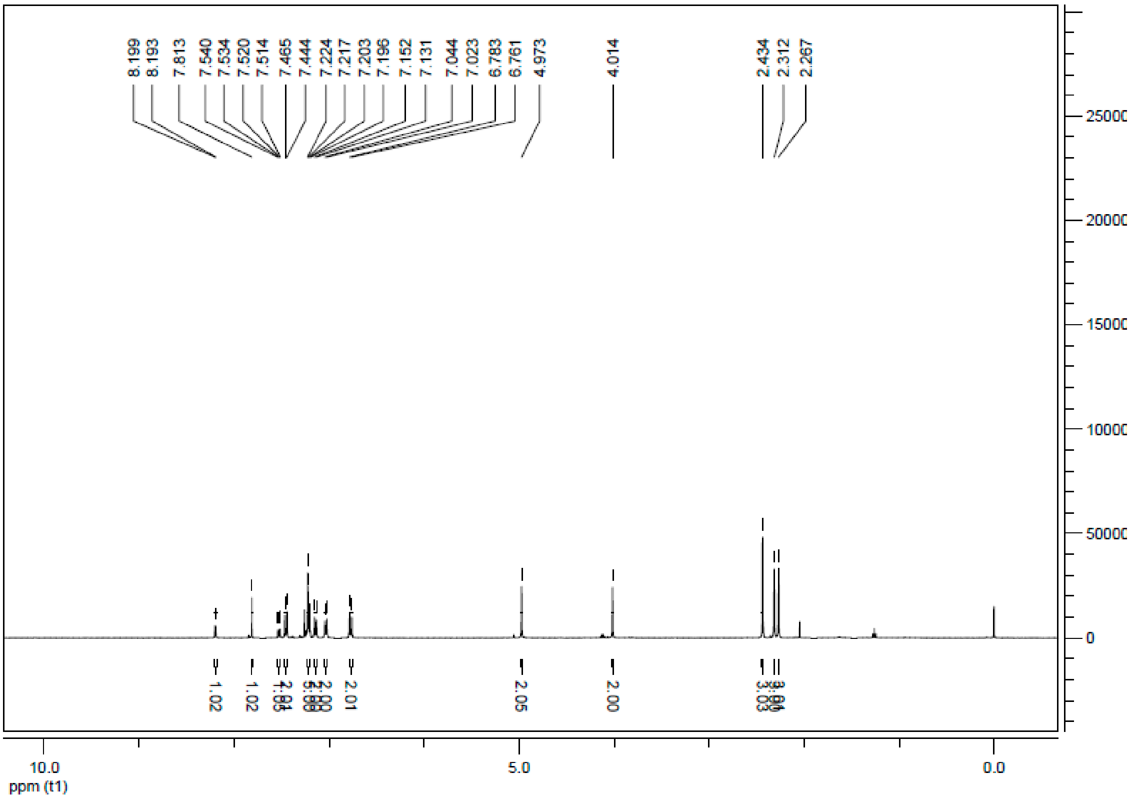

Figure S45. ^{1}H-NMR of compound **9w** (400 MHz, $CDCl_3$).

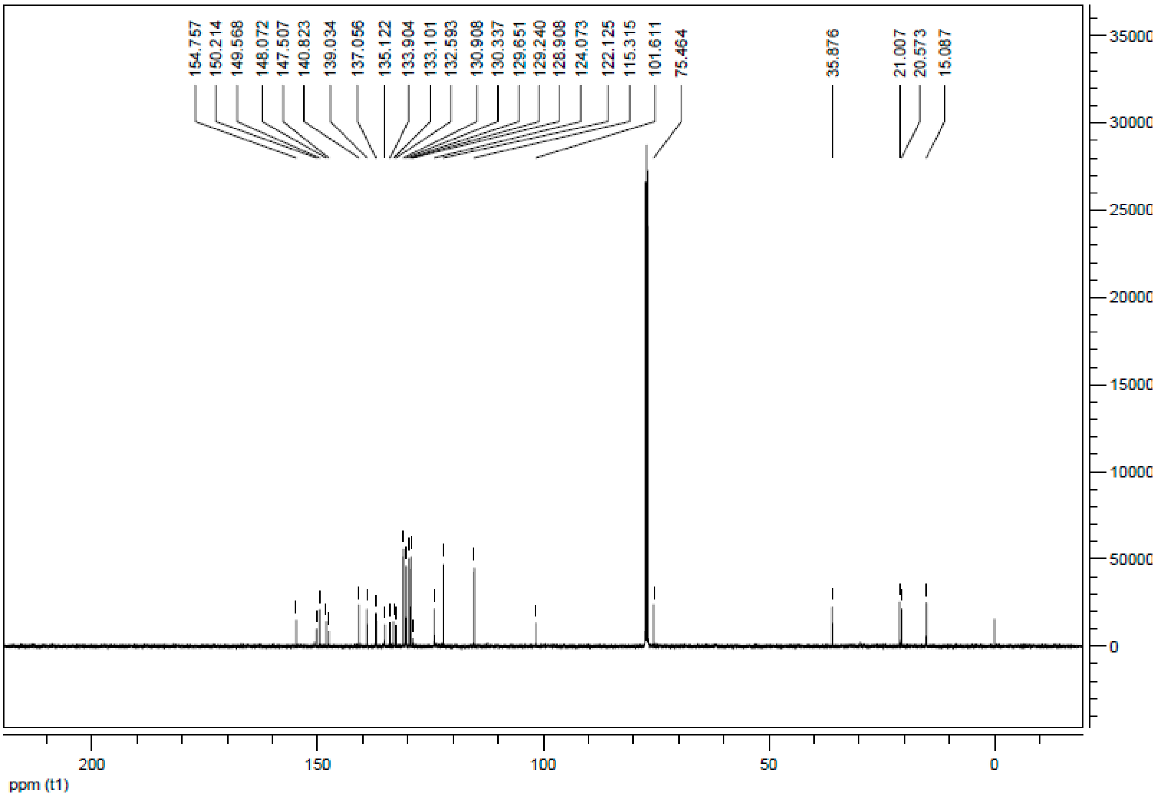

Figure S46. ^{13}C-NMR of compound **9w** (100 MHz, $CDCl_3$).
